# Caveolae Regulation of Mechanosensitive Channel Function in Myotubes

**DOI:** 10.1371/journal.pone.0072894

**Published:** 2013-08-30

**Authors:** Haixia Huang, Chilman Bae, Frederick Sachs, Thomas M. Suchyna

**Affiliations:** 1 Capital Medical University, Department of Physiology, Beijing, China; 2 SUNY at Buffalo, Department of Physiology and Biophysics, Buffalo, New York, United States of America; Emory University School of Medicine, United States of America

## Abstract

Mutations that lead to muscular dystrophy often create deficiencies in cytoskeletal support of the muscle sarcolemma causing hyperactive mechanosensitive cation channel (MSC) activity and elevated intracellular Ca^2+^. Caveolae are cholesterol-rich microdomains that form mechanically deformable invaginations of the sarcolemma. Mutations to caveolin-3, the main scaffolding protein of caveolae in muscle, cause Limbe-Girdle muscular dystrophy. Using genetic and acute chemical perturbations of developing myotubes we investigated whether caveolae are functionally linked to MSCs. MSC sensitivity was assayed using suction application to patches and probe-induced indentation during whole-cell recordings. Membrane mechanical stress in patches was monitored using patch capacitance/impedance. Cholesterol depletion disrupted caveolae and caused a large increase in MSC current. It also decreased the membrane mechanical relaxation time, likely reflecting cytoskeleton dissociation from the bilayer. Reduction of Cav3 expression with miRNA also increased MSC current and decreased patch relaxation time. In contrast Cav3 overexpression produced a small decrease in MSC currents. To acutely and specifically inhibit Cav3 interactions, we made a chimeric peptide containing the antennapedia membrane translocation domain and the Cav3 scaffolding domain (A-CSD3). A-CSD3 action was time dependent initially producing a mild Ca^2+^ leak and increased MSC current, while longer exposures decreased MSC currents coinciding with increased patch stiffening. Images of GFP labeled Cav3 in patches showed that Cav3 doesn’t enter the pipette, showing patch composition differed from the cell surface. However, disruption via cholesterol depletion caused Cav3 to become uniformly distributed over the sarcolemma and Cav3 appearance in the patch dome. The whole-cell indentation currents elicited under the different caveolae modifying conditions mirror the patch response supporting the role of caveolae in MSC function. These studies show that normal expression levels of Cav3 are mechanoprotective to the sarcolemma through multiple mechanisms, and Cav3 upregulation observed in some dystrophies may compensate for other mechanical deficiencies.

## Introduction

Cation permeable mechanosensitive channels (MSCs) are the most commonly observed type of MSCs in muscle cells [1], [2]. The protein identity of these channels is uncertain, but they are likely composed of heterogeneous channel types including TRP and Piezo channels and ACh receptors [3]–[10]. The mechanosensitivity of these channel types transduces changes in membrane tension into excitatory inward cation currents. The physiological role of these currents is not well understood, but is believed to play a role in myofiber development [11] and remodeling of the cortical cytoskeleton and myofibrils [2], [12], [13]. Dysregulation of MSCs can lead to increased Na^+^ and Ca^2+^ influx which has been implicated in the pathogenesis of multiple forms of muscular dystrophy [1], [14], [15].

Sarcolemma forces generated by muscle movement are buffered primarily by two specialized structures. The first is the dystroglycan complex (DGC) [16] that transmits forces from the extracellular matrix through intramembrane proteins to the cortical cytoskeleton. Mutations that lead to malformation of this complex have significant effects on cortical mechanics [2], [17] and MSC activity [1], [2], [18]. The second consists of membrane microdomains called caveolae that provide expandable membrane reservoirs when tension increases [19]. Caveolae are 50–150 nm membrane involutions that splay outward with membrane tension [20], [21]. The caveolae bilayer is a cholesterol/sphingosine rich domain with caveolin-3 (Cav3) protein intercalating into the inner leaflet. Cav3 helps stabilize the curvature of the domain, and acts as scaffolding for association of numerous signaling and cytoskeletal adaptor proteins [22], [23]. Mutations of Cav3 can lead to Limb Girdle muscular dystrophy type 1C and other muscle pathologies [24], [25]. Most caveolinopathies are marked by a reduced Cav3 surface expression due to sequestration of the protein in the Golgi. In addition, both overexpression and deficiency in Cav3 expression affects myotube development and causes disorganization of the cortical cytoskeleton such as the DGC [26] and mislocalization of signaling molecules [27], [28].

MSCs are sensitive to physical properties on the bilayer including factors that vary between lipid ordered (L_o_) membrane domains and the surrounding disordered lipid [29], [30]. Caveolae domains differ from the surrounding bilayer in curvature, sphingolipid/cholesterol composition and bilayer thickness [31]. In addition, caveolae have a unique protein composition and associations with the cortical cytoskeleton [15]. Many channel types, that are known to localize to caveolae [23] and to interact directly with Cav3 [32], have been indirectly associated with MSC activity in myofibers. For example, TRPC1 channels associate with Cav3 and its expression is elevated in myofibers from the *mdx* mouse (a Duchene muscular dystrophy model) linking them to the elevated intracellular Ca^2+^ [3]. TRPC1 channels have been reported to be mechanosensitive [33], although this has been disputed [34].

Regardless of the channel type, caveolae structure, composition, and mechanical properties make them prime candidates for controlling MSC function. To test this hypothesis, we manipulated caveolae in myotubes using biochemical and genetic tools and assessed the effects on MSC activity and cortical mechanics in patches and in whole cell recordings. Although many techniques have been used to investigate MSCs (e.g. cell swelling, applying pressure with glass probes, magnetic beads, flow induced shear stress), the highest resolution method for evaluating the direct link between membrane stress and channel function is the patch. This is because other methods have defined stimuli but do not measure the local stress. Patch capacitance measurements have been particularly useful in defining the mechanical kinetics of the patch membrane. The mechanical relaxation is significantly slower than the pressure stimulus due to the viscoelastic properties of the supporting cytoskeleton [2], [35]–[37]. Using the patch we can distinguish between changes in channel sensitivity to stress and changes in membrane mechanics. We have shown that patch formation itself disrupts normal membranes causing changes in composition and exerting large resting stress (∼20% of lytic tension) due to adhesion to the glass. However, the basic properties of MSCs are maintained in the patch as shown by the similarity of activation and inactivation kinetics of Piezo MSCs between patches and whole cell currents activated by probe displacement [10], [38].

A clear trend emerged from this study showing that greater caveolae disruption leads to increased MSC currents and more rapid patch mechanical relaxation. We also measured the effect of temperature on MSC activity since temperature is known to change the physical properties of L_o_ domains [39], [40]. We measured the localization of fluorescently labeled Cav3 and other caveolae components in the patch. Finally, whole-cell currents also increased with caveolae disruption, though there were differences between the whole-cell and patch MSC current properties.

## Methods

### Myotube Cultures

This study was carried out in strict accordance with the recommendations in the Guide for the Care and Use of Laboratory Animals of the National Institutes of Health. Mice were dispatched by cervical dislocation according to a protocol approved by the SUNY at Buffalo Institutional Animal Care and Use Committee (Project # PGY28025N). All efforts were made to minimize suffering. Myocytes were isolated from C57-BL10 mice ages 6–12 weeks as previously described [2]. Myotube development usually began between 3–5 days post-plating. For electrophysiological analysis myotubes between 10–20 days post-plating were used. Cells were transfected on day 2 after plating with 100 µl of serum free DMEM containing 0.7 µg of DNA and 4 µl of Fugene-6 reagent from Roche Applied Sciences (Indianapolis, IN). Cells were incubated with DNA for 48 hrs in high growth media before switching to differentiation media on day 4 after plating. Exposure of myocytes to the Fugene 6/DNA transfection mixture strongly induced myotube formation (Fig. S1). Exposure to Fugene-6 alone also induced myotube formation, but not as effectively as DNA combined with the Fugene 6 reagent. Without transfection few myotubes formed and most were poorly developed (see discussion).

### Electrophysiology

**Figure 1:**
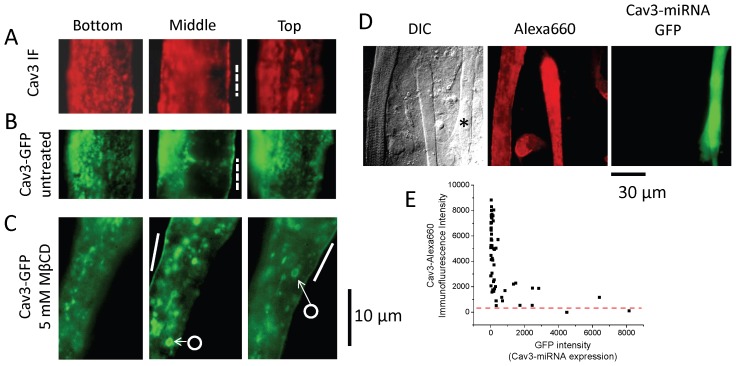
Genetic and chemical manipulation of caveolae. Immunofluorescence labeling of endogenous Cav3 expression (Alexa 660) was compared to the distribution of exogenous expression of Cav3-GFP in myotubes. (A) Images of Alexa 660 immunolabeled Cav3 shows focal planes at the bottom (near the coverslip), the middle and the top of the cell. Cav3 has a punctate distribution at the cell surface (indicated by –-) and a reticulate internal structure. (B) Exogenously expressed Cav3-GFP shows the same patterns of distribution. (C) Cav3-GFP expressing cells treated for 1 hr with 5 mM MβCD at 37°C makes the surface distribution homogeneous (indicated by –––) and the internal proteins become associated with large vesicular structures (indicated by O). The image stacks showing all z-sections are shown in Movies S1, S2, and S3. (D) Cav3-miRNA inhibits endogenous expression of Cav3 protein as shown by Alexa 660 immunofluorescence. DIC images show several myotubes. The myotube indicated with * has high GFP expression and low endogenous Cav3 expression compared to untransfected myotubes in the image. (E) Cells transfected with Cav3-miRNA showed significantly lower endogenous Cav3 expression than untransfected cells. The red dashed line shows the average Alexa 660 intensity of untransfected cells labeled without the primary antibody.

Patch clamping used an Axopatch 200B (Axon Instruments, CA) and experimental protocols were controlled by Axon Instruments pClamp10 software via a Digidata 1322A acquisition system. Currents were sampled at 10 kHz and low-pass filtered at 1 kHz through the 4 pole Bessel filter on the Axopatch 200B. All potentials are defined as membrane potentials with respect to the extracellular surface. Electrodes were painted with Sylgard 184 (Dow Corning Corp. Midland MI) and fire polished. Electrodes were filled with KCl saline (KCl 140 mM, EGTA 2 mM, MgCl_2_ 2 mM, HEPES 10 mM, pH 7.3) and had resistances ranging from 4–8 MΩ. Bath saline consisted of NaCl - 140 mM, KCl - 5 mM, CaCl_2_ - 2 mM, MgCl_2_ - 0.5 mM, glucose - 6 mM and HEPES - 10 mM, pH 7.3. Cells had a resting membrane potentials ranging from −50 to −70 mV. At a pipette potential of −60 mV the patch was near the reversal potential for the cation selective channels. Hydrostatic pressure was applied to the pipette by an HSPC-1 pressure clamp (ALA Scientific Instruments, NY) controlled by pClamp software. A typical experiment started with −30 mmHg pressure steps (MSC activation threshold) and proceeded to −90 mmHg (near rupture) in −20 mmHg increments. At each pressure we applied 10–15 pressure steps at −60 and +60 mV, and the mean conductance and current were recorded respectively (see Figure 1). Ensemble average currents of a particular condition were made by averaging the mean currents at −70 mmHg across multiple patches. Off-line data analysis was performed with Clampfit and Origin 8.5 software.

### Capacitance and Conductance Measurements

Patch capacitance and conductance were measured as previously described [2], [36]. We used a Princeton Applied Research dual phase lock amplifier (PLA) model EG&G 5207 (Oak Ridge, TN) to apply a 2 kHz carrier signal of 20 mV RMS to the Axopatch amplifier external input. To view simultaneous channel activity, we suppressed the carrier signal with the transient balance and used a low pass filter setting below the carrier frequency to record the current from the front panel scaled output on the Axopatch. The signal from the rear output of the patch clamp, containing the unfiltered carrier, was fed to the PLA. We first nulled the carrier signal from the patch clamp using the pipette capacitance compensation. Then to provide a “pure capacitance” reference signal, we unbalanced the fast transient amplitude compensation, locked the PLA to that signal, and then re-nulled the transient compensation. The in-phase signal from the PLA (the real part of the patch impedance, the conductance) was generally a mirror of channel activity. The signal 90° out of phase represented changes in patch capacitance.

### Indentation Assay

Whole-cell experiments used the Axopatch 200B amplifier (Axon Instruments, CA) in whole-cell mode. Patch pipettes had a resistance of 2–5 MΩ when filled with the same solutions as used in patch experiments. The extracellular solution was also identical to the one described above. Whole-cell mechanical stimulation utilized a fire-polished glass pipette (tip diameter of 2–4 µm) positioned at an angle of 30° with respect to the coverglass. To apply indentations the probe was coarsely positioned ∼20 µm from the cell. From that position, an 8–10 µm trapezoidal downward movement was driven by a piezoelectric stage (either a P-280.20 XYZ NanoPositioners, Physik Instrumente or a Burleigh PZ-301, Thorlabs, Newton, NJ) controlled by the QUBIO (www.qub.buffalo. edu). The cell indentation depth (probe baseline depth) was controlled by a computer-controlled micromanipulator with 40 nm resolution (MP-285, Sutter Instruments Co.) using LabVIEW code. The starting baseline position was defined as the depth at which the probe first visibly deformed the cell during the plateau phase of the stimulus. The probe velocity was 0.14 µm/ms during the upward and downward movement, and the stimulus was kept constant for 300–500 ms at the plateau. Currents were recorded at a holding potential of −60 mV.

### Chemicals and Vector Constructs

A-CSD3 peptide was synthesized with the 19 aa scaffolding domain of Cav3 (DGVWKVSFTTFTVSKYWCYR-amide) fused to the c-terminus of the 16 aa antennapedia translocation domain (RQIKIWFQNRRMKWKK). A-CSD3 peptide was made by (CSD Bio-WORLD, Dublin, OH) and dissolved in DMSO at 10 µg/µl prior to dilution in saline for testing.

BODIPY-cholesterol and BODIPY FL C_5_-lactosylceramide complexed with BSA were obtained from Invitrogen (Carlsbad, CA). A stock solution of 1∶22.5 mg ratio of BODIPY-cholesterol: MβCD was made in bath saline and incubated at 42°C for 1 hr while vortexing every 15 min. 25 µl of the stock solution was added to 1 ml of saline and vortexed to make a 25 µg/µl working solution. Cells were incubated in working solution for 3–5 min at 37°C and then washed twice with bath saline. For sphingosine localization, myotubes were incubated for 20 min at 21°C in a working solution of 5 µM BODIPY FL C_5_-lactosylceramide dissolved in bath saline and washed four times with bath saline.

The cDNA clone of N-terminal GFP fusion to Caveolin-3 was constructed in a modified pNGFP-EU vector generously provided by Eric Gouaux (Columbia University). We also used Caveolin-3 in the pIRIS expression system that co-expresses GFP. Caveolin-3 miRNA vector was a bicistronic clone from Invitrogen (Carlsbad, CA) coding for two miRNA sequences starting at nucleotide sites 407 and 638.

### Video Microscopy and Analysis

Patches were visualized on a Zeiss Axio Observer Z1 inverted microscope with DIC optics, motorized Z-axis and DefiniteFocus Z-axis positioning (Oberkochen, Germany). The microscope was outfitted with a Ludl (Hawthorne, NY) LEP 96S108-Z3-LE2 motorized X-Y stage. The patch pipette was aligned perpendicular to the axis of the Wollaston prisms and the pipette approached the cover slip at ∼15° from horizontal. The imaging optics were a Zeiss 63×1.4 NA oil immersion objective and a condenser made of a 40×0.8 NA water immersion objective. The objective temperature was controlled with a Bioptechs (Butler, PA) objective heater and the solution immediately above the objective maintained a temperature within 1°C of the objective temperature set on the controller. Images were collected with an Andor iXon DV897 camera (South Windsor, CT) mounted on a 4× lens. Microscope components were controlled by MicroManager software [41]. BODIPY and GFP fluorescent images were acquired using a narrow band GFP filter set (Chroma Technology, Bellows Falls, VT) with excitation band 465–490 nm and emission band 500–520 nm. Image processing and analysis used ImageJ software (rsb.info.nih.gov/ij/).

### Ca^+2^ Fluorescence Imaging

Myotube cultures were incubated for 15 minutes in bath saline containing 1 µM Fluo4 AM and then in bath saline for another 15 minutes. Experiments were performed at 21 and 37°C. For experiments performed at 37°C a heated lid was placed over the coverslip holder to maintain the entire bath at the desired temperature. Fluo4 fluorescence was excited using the narrow band GFP filter set described above. Test substances were applied by whole bath perfusion. Multiple cells were imaged over time using the X-Y position list and the MultiD acquisition functions in MicroManager.

### Statistical Analysis

Graphed average values show means ± se. Statistical differences for the different conditions were evaluated with a two sample t-test in Origin 8.5 software using 5% (**) and 10% (*) significance levels and assuming non-equal variance for the data sets.

## Results

### Myotube Differentiation and Manipulation of Caveolae

MSC activity in patches is sensitive to the developmental stage of the myotubes [1]. Activity can be affected by the channel concentration and the developmental expression pattern of structural proteins that control membrane tension. For example endogenous caveolin-3 expression begins early during myotube development (2–3 days in culture [42]) while dystrophin expression is delayed (11–17 days in culture [43]). In our cultures we observed that, although myotubes appear within 3–5 days after plating, larger myotubes with better defined sarcomeres appear in older (10–20 days) cultures (Fig. S2). For consistency, only mature myotubes were used (10–20 days).

Three methods were initially employed to modify caveolae: Cav3 over-expression, inhibiting Cav3 expression, and caveolae disruption by cholesterol depletion. We transfected myocyte cultures with either a Cav3-GFP fusion protein to overexpress the protein or with miRNA to knockdown Cav3 mRNA levels. Immunolabeling of endogenous Cav3 showed a punctate distribution on the sarcolemma and reticulate structures penetrating deeper into the cytoplasm (Fig. 1A and Movie S1). This is consistent with published immunofluorescence studies in myotubes [44]. Expression of a Cav3-GFP fusion protein showed a similar distribution to the endogenous labeled protein (Fig. 1B and Movie S2). A similar fusion protein, Cav3-YFP, was recently shown to induce caveolae formation in myocyte membranes, and these caveolae showed dynamic rearrangements when stretched [21]. When cholesterol was depleted with 5 mM methyl-β-cyclodextrin (MβCD) for 45 min at 37°C, the Cav3-GFP fluorescence became homogeneous on the sarcolemma, and the internal reticulate structures became vesiculated (Fig. 1C and Movie S3). MβCD treatment caused a similar redistribution of Cav1-GFP in HELA and MDCK cells [45], and loss of caveolae structures from the membrane surface of smooth muscle cells [46]. MβCD treated myotubes remained elongated and contracted with depolarization for more than an hour after treatment. Transfection with Cav3 miRNA produced an ∼10 fold decrease in endogenous Cav3 expression as shown by immunofluorescence (Figs. 1D and E).

### The Patch Assay and MSC Characterization

**Figure 2:**
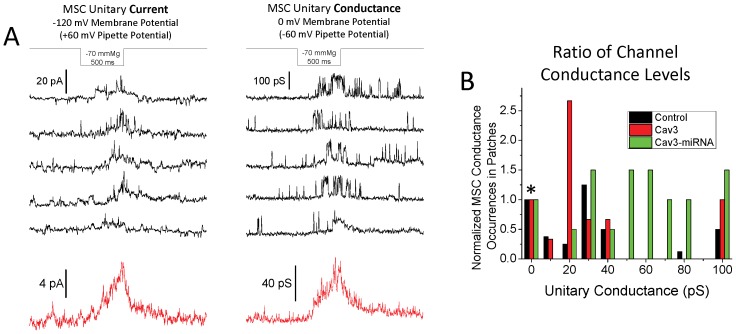
Myotube MSC patch currents. (A) Typical recordings from a myotube cell-attached patch. The first panel shows the four representative single channel current records of MSCs at −120 mV, and the second panel shows the single channel conductance records at 0 mV. More background channel activity during the resting phase between pressure steps is noticeable and the kinetics of the channel openings changes significantly between the two voltages. Below the individual records are red traces showing the mean MSC current from typically 5–15 pressure steps at that voltage and pressure. The kinetics of the average responses were similar at both voltages so only the average currents are used in subsequent figures. (B) Shows the main maximum conductance levels observed for patches from control, Cav3-GFP and Cav3-miRNA expressing cells. The main conductance states were determined by either all points histogram analysis or by directly measuring the height of the most common channel transition events of the records during the pressure steps. Often there were two maximum channel conductance levels identified within a single patch that were distinguished from substates based on kinetic differences. Larger conductance channels usually had much shorter open states life times. The number of conductance states for the three different conditions were normalized to the number of patches that showed no MSC currents (*) to more proportionately illustrate the differences among groups with different numbers of patches. The number of MSC positive patches for all conditions was >70%. The ratio of patches displaying MSCs/total number of patches was: Control - 20/28, Cav3-GFP - 13/16, Cav3-miRNA –15/17. Patches from Cav3-miRNA expressing cells showed a distinct shift to channels of higher conductance.

Figures 2A shows representative single channel current and conductance recordings of MSCs in cell-attached patches at two different membrane potentials. Both records show channel openings as upward deflections for ease of comparison to each other and to the capacitance recordings. The average current/conductance is shown below in red. The average resting potential of myotubes was about −60 mV as measured when breaking into whole cell mode. The first group of ***current*** recordings at ∼−120 mV membrane potential normally showed significant background channel activity at 0 mmHg. The identity of these background channels was not determined although they may be MSCs activated by the resting tension in the patch [35]. However, the presence of MSCs was clearly observed during the pressure steps in both the single and averaged current records. The patch was most stable at ∼0 mV membrane potential during multiple pressure step protocols. However, with no net driving potential, channel activity is difficult to assess in the current recording. At this voltage the in-phase signal (***conductance***) from the phase lock amplifier was used [36]. MSCs in cell-attached mode typically do not inactivate at either voltage as shown in the average current/conductance records and in previous reports [2], [47]. Typically the background channel activity is minimal at 0 mV which gives a clearer assessment of the MSC conductance levels. Even at this voltage, most patches display multiple MSC conductance levels (see different examples in Fig. S3) suggesting the presence of heterologous channel types. In addition, MSCs often enter subconductance levels [36], [47] making it difficult to assign a specific conductance to the channels.

From histogram analyses we estimated the dominant single channel current/conductance levels from most patches. Figure 2B shows the range and distribution of these maximum conductance levels observed in individual patches from control cells, and cells expressing either Cav3-GFP or Cav3 miRNA. The conductance levels for each group were normalized to the number of patches that did not show any MSC activity (denoted by *). While >70% of patches in all three groups showed MSC currents, Cav3-miRNA patches had channels shifted toward larger conductances (50–100 pS) and Cav3-GFP patches had a high percentage of channels in the 20–30 pS range. Every patch from MβCD treated cells (not shown in the histogram) had MSC activity with the majority of patches (6 out of 9) showing channels in the 20–30 pS range. For all conditions, the channels in the 50–100 pS range tended to have more discrete open states and faster kinetics (see Fig. 2A, conductance records).

The heterologous nature of the MSCs in myotubes made identification of the channel types difficult. The postulated identity of the MSCs has been published multiple times (see discussion). For our studies, we averaged across the heterogeneity which is important for understanding the role of caveolae in the regulation of the net signaling system. “Ensemble averaged” patch records are used for the remaining figures that were created by averaging across (≥15) patches.

Temperature will modulate the size and dynamics of L_o_ membrane domains [48] and the trafficking of caveolae [49], so we compared patch recordings made at 37°C and at 21°C (our normal recording temperature, Fig. S4). The MSC current (ΔIp) was about two fold larger at 37°C than at 21°Cfor all pressures (Fig. S4A and B). The increase corresponds to a Q_10_ of 1.6, consistent with the permeation rate increase expected for ion channel pores [50]. We used patch capacitance measurements (ΔCp) to monitor changes in the dome area and parameterized the mechanical relaxation kinetics at both temperatures by curve fitting (Fig. S4A). In essence the dome area change is a measure of the cytoskeletal mechanics because the kinetics of the cortex are dominated by the cytoskeletal associations [2], [35]. The magnitude of the ΔCp appeared to be temperature independent when simply measuring the area under the capacitance curve during the 500 ms pressure step (Fig. S4C). However, exponential fits to the data clearly showed significant differences in the relaxation rate and steady state magnitude (Fig. S4D). The rate and magnitude at 21°C were similar to those previously reported for normal myotube patches [2]. However, at 37°C the rate unexpectedly decreased by >40% (∼200 ms slower) and the estimated steady state capacitance increased by >40% (∼13 fF greater). The slower rate at 37°C suggests that the cytoskeleton became more viscous. These mechanical differences and the larger currents prompted us to focus our attention on the more physiologically 37°C.

The response to 500 ms stimuli rarely reached steady state so we had to rely on curve fitting to approximate the steady state. To determine whether our fitting analysis was a good representation of the mechanical relaxation kinetics we compared the 500 ms ΔCp to the response from longer stimuli that should reach steady state (Fig. S5A). Even with 2500 ms stimuli, the response didn’t reached steady state and also did not return to the baseline due to patch creep [35], requiring a second order exponential for acceptable fits. This was not the case for shorter 500 ms steps that usually returned to baseline during the time between steps. Comparing ΔCp at 500 ms and 2500 ms in the same patches showed that the relaxation rates predicted from fits to the 500 ms curves were about 20% lower than the 2500 ms estimates (Fig. S5B). In addition the predicted steady state ΔCp for the 500 ms steps was ∼15% lower than the measured ΔCp from 2500 ms steps. Thus, due to the nonstationary response of longer stimuli we found that estimating the steady state capacitance from fits to the 500 ms response was more reliable over a range of pressures.

### Manipulation of Caveolin Expression and Distribution Affects MSC Gating

**Figure 3:**
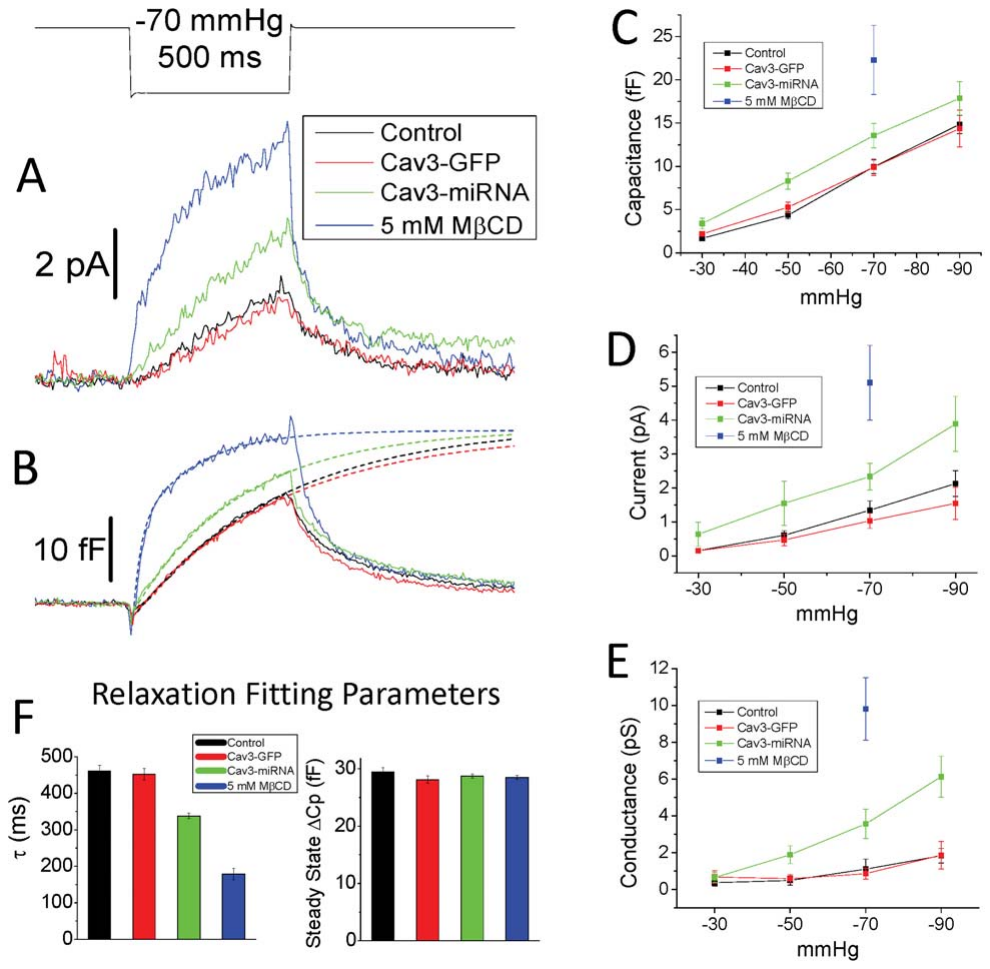
MSC patch currents increase when caveolae are disrupted or reduced. ΔCp kinetics indicates cytoskeletal interactions with the membrane. Reduced interactions are normally associated with a more rapid patch mechanical relaxation rate. Ensemble averaged MSC currents at −110 mV (A) and patch stress represented by ΔCp (B) are shown for control (n = 27) Cav3 overexpression (n = 16), Cav3 reduction by inhibitory RNA (n = 16, Cav3-miRNA) and cholesterol depletion (n = 9, 5 mM MβCD). The exponential fits to the rising phase relaxation curves of the ensemble capacitance records are shown as dashed lines through the data and extended out to 1.2 seconds. Ensemble averaged ΔCp (C), channel current at −120 mV (D) and patch conductance at 0 mV (E) over the range of pressures studied are shown (SEM error bars). Patches from MβCD treated cells were fragile so data was collected at −70 mmHg first, before patch rupture which often occurred before other pressures could be sampled. Cav3 overexpression had no effect on the MSC activity or patch mechanics suggesting saturation of caveolae components. Patches from cells expressing Cav3-miRNA showed a significant increase in MSC current, patch conductance and increased ΔCp where the faster relaxation suggested a more compliant patch (see Fig. S4B for statistical analysis). Patches from cells treated with 5 mM MβCD showed the largest increase in all parameters measured. The parameters from the ΔCp curve fits are shown in (F). Either first or second (MβCD) order exponentials were used and the fitting data shows that the relaxation rates decreased as patch current increased, but the predicted steady state ΔCp stayed the same. This shows that factors that disrupt caveolin do not change patch size but do affect the relaxation rate.

Changes in caveolin expression on the ensemble patch current and capacitance records are compared in Figures 3A and B at −70 mmHg. Figures 3C, D and E show plots of the mean capacitance, current and conductance during 500 ms pressure steps over the range of pressures studied. We did not see the response saturate over this pressure range, and pressures larger than −90 mmHg usually resulted in patch breakdown. While these pressures are larger than reported elsewhere [1], they are consistent with our previous reports [2]. We believe the lower pressures required by other labs are related to differences in the glass or saline compositions used that can affect the resting tension in the patch dome and the patch mechanical properties during stimulation [35], [51].

Cells overexpressing Cav3 showed nearly identical MSC and patch mechanical responses as control cells suggesting the system is saturated at normal expression levels. However, inhibiting expression of Cav3 with Cav3-miRNA nearly doubled the MSC current and conductance, and produced a significant increase in the magnitude of ΔCp (See Fig. S6B for statistical analysis). Disrupting caveolae by cholesterol depletion tended to cause patches to become flat or convex toward the inside of the pipette showing a loss of cytoskeletal association that create forces normal to the dome surface [35]. The patches also became fragile so that usually only one set of pressure steps could be obtained before rupture. For this reason we show only the data points at −70 mmHg. Disrupting the caveolae (and likely other membrane domains) by this cholesterol depletion increased MSC currents and conductance levels 5 and 10 fold respectively, and doubled the ΔCp. The correlation of increased channel activity with increased patch capacitance supports the idea that MSC are gated by bilayer tension, since activity increases as parallel elastic cytoskeletal elements are disrupted [35], [36], [52].

The mechanical relaxation rates for controls, Cav3-GFP and Cav3-miRNA at −70 mmHg were modeled with a single exponential (Fig. 3F). However, the response for patches from MβCD treated cells was more accurately fit with a second order exponential suggesting two independent processes; likely a combination of changes in membrane physical properties and peeling of the seal region. The rates of all processes controlling membrane area change are likely significantly retarded with greater cytoskeletal associations. Cav3-GFP produced no change in the relaxation kinetics compared to controls. Cav3-miRNA patches had a faster relaxation time but the estimated steady state level was unchanged suggesting changes in viscosity rather than elasticity. This was similar for patches from MβCD treated cells that showed a rapid relaxation rate but the same steady state level. To compare MβCD relaxation rates in Figure 3F to the other treatments we used the slower time constant (τ_1_ = 16±1.3 ms, τ_2_ = 164±11 ms). The similarity of the steady state capacitance levels likely represents consistency in the patch diameters (3.41±0.06 µm – control, 3.45±0.11 µm – Cav3-GFP, and 3.32±0.06 µm – Cav3-miRNA) suggesting that kinetic differences are the result of varying cytoskeletal associations. However, patches from MβCD treated cells had 35% smaller diameter (2.7±0.13 µm) due to sealing closer to the tip. The larger specific ΔCp (stress induced ΔCp/dome area) of MβCD patches is likely due to changes in the extent of seal peeling and/or the flow of lipids into the dome [35], [53]. These parameters could both be affected by the reduced cytoskeletal associations or the modified bilayer composition in these patches.

We previously showed that patches from *mdx* myotubes missing the protein dystrophin had a significant increase in the “background” MSC activity at rest [2]. In the present experiments, Cav3 depletion or disruption caused an increase in MSC current during the pressure step, but little change in the background current under the different conditions (Fig. S6C) suggesting that caveolin and dystrophin control MSC current by different means.

### Specific Disruption of caveolin-3 Interaction

**Figure 4:**
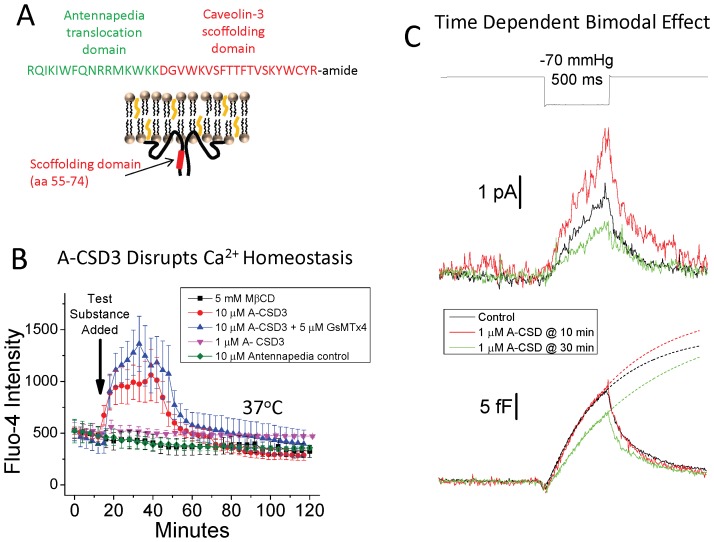
Inhibition of Cav3 binding affects Ca^2+^ homeostasis, cytoskeletal-membrane interactions and MSC activity. A-CSD3 affects Ca^2+^ fluxes in a manner different from cholesterol depletion, and at 1 uM the peptide has a time dependent bipolar potentiating/inhibitory effect on MSC activity in patches. (A) Illustrates the two amino acid sequences joined to form the A-CSD3 peptide and the location of the scaffolding domain in Cav3. (B) We loaded myotubes with Fluo4-AM and monitored Ca^2+^ levels during application of test substances. Images were taken every 3–5 minutes from 15–20 myotubes per coverslip. Only myotubes with no overlying cells (direct bath exposure) were chosen for monitoring. Cells that were beneath other cells (fibroblasts, myocytes and other myotubes) were either unaffected or showed long delays with muted responses to test substances. This demonstrates that A-CSD3 provides a rapid means of disrupting Cav3 interactions that clearly have central role in regulating sarcolemma integrity. Addition of 5 mM MβCD did not induce a Ca^2+^ response. However, 10–15 µM A-CSD3 rapidly produced a large Ca^2+^ elevation that was not blocked by the MSC blocker GsMTx4. As a control 10 µM antennapedia peptide alone was added to the cells and had no effect. Application of 1 µM A-CSD3 control peptide produced a relatively small but sustained Ca^2+^ elevation. (C) Ensemble averaged MSC currents and ΔCp at −70 mmHg were measured from control (untransfected) myotubes at ∼10 (n = 14) and ∼30 (n = 16) min after addition of 1 µM A-CSD3. The exponential fits to the rising phase of the stimulus relaxation are shown as dashed lines. At 10 min, the MSC current increased with no change in ΔCp. After 30 min the MSC current decreased and there was a corresponding decrease in ΔCp and an increase in the relaxation rate. See Fig. S4B for statistical analysis of ensemble MSC current changes. Thus acute disruption of Cav3 interactions can affect MSC currents independent of the mechanical effects on the membrane.

Genetic manipulation of caveolin expression has long term developmental effects on the expression levels of proteins in the sarcolemma [25]. Cholesterol manipulation affects the physical properties of the membrane [54] and the association and distribution of many cortical cytoskeletal proteins to the membrane [55], [56]. Thus, the manipulations used above could affect MSC activity in multiple ways. So we sought a means to acutely and specifically inhibit Cav3 interactions to help determine its role in MSC regulation. Caveolin possesses a 19 amino acid N-terminal scaffolding domain (CSD) which is involved in caveolin oligomerization and interactions with other proteins [23]. Rapid and specific inhibition of CSD interactions has been achieved by using a fusion protein containing the caveolin-1 CSD and the 16 aa antennapedia domain [57] that allows translocation across the plasmalemma for external applications. In the cytoplasm, this fusion protein inhibits heteromeric and homomeric protein interaction with this site on caveolin. We made a similar peptide by fusing the antennapedia peptide to the caveolin-3 scaffolding domain (A-CSD3) as shown in Figure 4A. The CSD3 domain differs in only five amino acids from CSD1, four of which are conservative.

To show that A-CSD3 peptide affects Cav3 interactions we initially tried to duplicate previous studies done with the anntenapidia-Cav1 peptide in endothelial cells [58], [59] by applying 10–20 µM A-CSD3 to myotubes at 37°C. In those previous studies, a significant cation influx was generated by anntenapidia-Cav1 peptide after a 4 hr incubation. Ca^2+^ influx would also be a likely consequence of increased cation MSC activity, and so we loaded cells with Fluo4 Ca^2+^ indicator and monitored the fluorescent intensity changes.

At 10–15 µM, A-CSD3 caused a rapid (∼5 min) Ca^2+^ influx coinciding with a strong disruptive contraction (Fig. 4B and Movie S4). This was followed by complete dissociation of the sarcolemma from the cytoskeleton forming large blebs of membrane and disruption of the sarcomeric structure. Most of the other cells in the cultures (fibroblasts, myocytes, etc.) were unaffected. This confirmed the internal accessibility of the A-CSD3 peptide and the central role of Cav3 in regulating myotube channel activity and sarcolemma integrity. In contrast, the majority of cells treated with 5 mM MβCD showed no change in Ca^2+^ levels or morphology after 2 hrs (Fig. 4B and Movie S5). Some myotubes in 5 mM MβCD were disrupted and exhibited a slow Ca^2+^ leak but did not release significant amounts of membrane as did the A-CSD3 treated cells. Removal of Ca^2+^ from the media during A-CSD3 treatment significantly delayed the Ca^2+^ influx and diminished its magnitude (Fig. S7) suggesting that influx was the main contributor to the Ca^2+^ increase. However, even in low Ca^2+^ the cells still swelled and released sarcolemma within minutes after exposure, suggesting that A-CSD3 induced Ca^2+^ influx and membrane-cytoskeletal dissociation involve distinct mechanisms. To explore whether the Ca^2+^ influx was a result of cationic MSC activation or voltage dependent calcium channel activation we combined A-CSD3 treatment with either 5 µM GsMTx4 (an MSC inhibitor, Fig. 4B) or a cocktail of VACC inhibitors (Fig. S7). Neither blocked the A-CSD3 effects, suggesting additional Ca^2+^ pathways. Treating the myotubes with 10 µM antennapidia peptide alone had no effect on Ca^2+^ levels or cell structure (Fig. 4B).

When the concentration of A-CSD3 was reduced to 1 µM, the cells were not disrupted and exhibited only a weak sustained Ca^2+^ influx for over 2 hours (Fig. 4B). However, after 15–20 min of exposure, patch formation became slower and greater suction was required for sealing. This situation was reminiscent of patch formation at high salt concentrations suggesting cytoskeletal stiffening [35]. Patches from these cells displayed a bimodal time-dependent response (Fig. 4C). After ∼10 min of exposure to 1 µM A-CSD3 there was a significant increase (∼60%) in MSC current (Fig. S6B), but no corresponding change in ΔCp, and only a small increase in the relaxation time (τ = 535±30 ms, steady state amplitude 34 fF). After 30 min there was a ∼50% decrease in MSC current coincident with a significant increase in the relaxation time (τ = 776±76 ms, steady state amplitude 32.3 fF). The longer relaxation times suggest increased cytoskeletal interactions with the patch dome and/or increased cytoskeletal stiffening through crosslinking [35], [60]. It is possible that the sustained low level Ca^2+^ leak triggered cytoskeletal modifications that caused the stiffening. Both time frames showed high levels of MSC activity (12 out of 14 patches at 10 min, and 13 out of 16 patches at 30 min), and both were dominated by channels with conductances in the 20–40 pS range (11 out of 14 patches at 10 min, and 11 out of 16 patches after 30 min).

**Figure 5:**
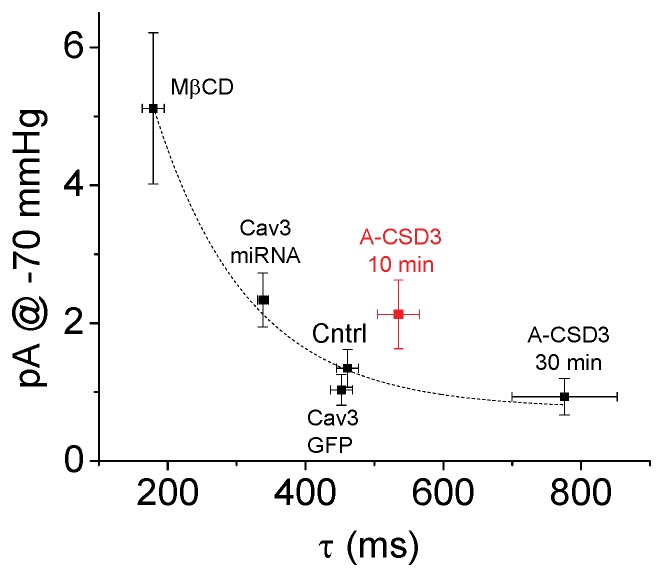
Increased relaxation rates correlates with decreased MSC current. Cav3 has significant effects on the membrane mechanics, as shown by the plot of the relaxation rates from curve fitting the ΔCp vs the average MSC current. In general as the patch relaxation rate increases the MSC current decreased. An exception was observed 10 min after treatment with 1 µM A-CSD3 (highlighted in red) where there was a slight increase in the relaxation rate, but an increase in MSC current.

When we plotted the relaxation rates for the different treatments vs the patch current at −70 mmHg (Fig. 5) we observed that MSC activity decreased with increasing membrane stiffness as might be expected when the cytoskeleton bears a larger fraction of the cortical stress. But as stated above, disrupting Cav3 interactions with A-CSD3 caused an increase in MSC activity that preceded the change in stiffness showing that Cav3 effects on MSC activity are complex.

### Patch MSC Characterization and Molecular Composition

Because the patch is formed from a bleb of membrane, its composition and structure are different from the unaltered cell surface [35]. Caveolae are known to be disrupted by membrane tension that is substantial during patch formation [19], [20]. Thus caveolae are not likely to remain intact in the blebbed membrane. Also due to the line tension at the caveolae perimeter [31], they may be less likely to contribute bilayer material to the patch blebs than the surrounding disordered lipids. But can caveolae reform in the patch dome after the seal is formed since dome structure can be highly irregular [35]?

**Figure 6:**
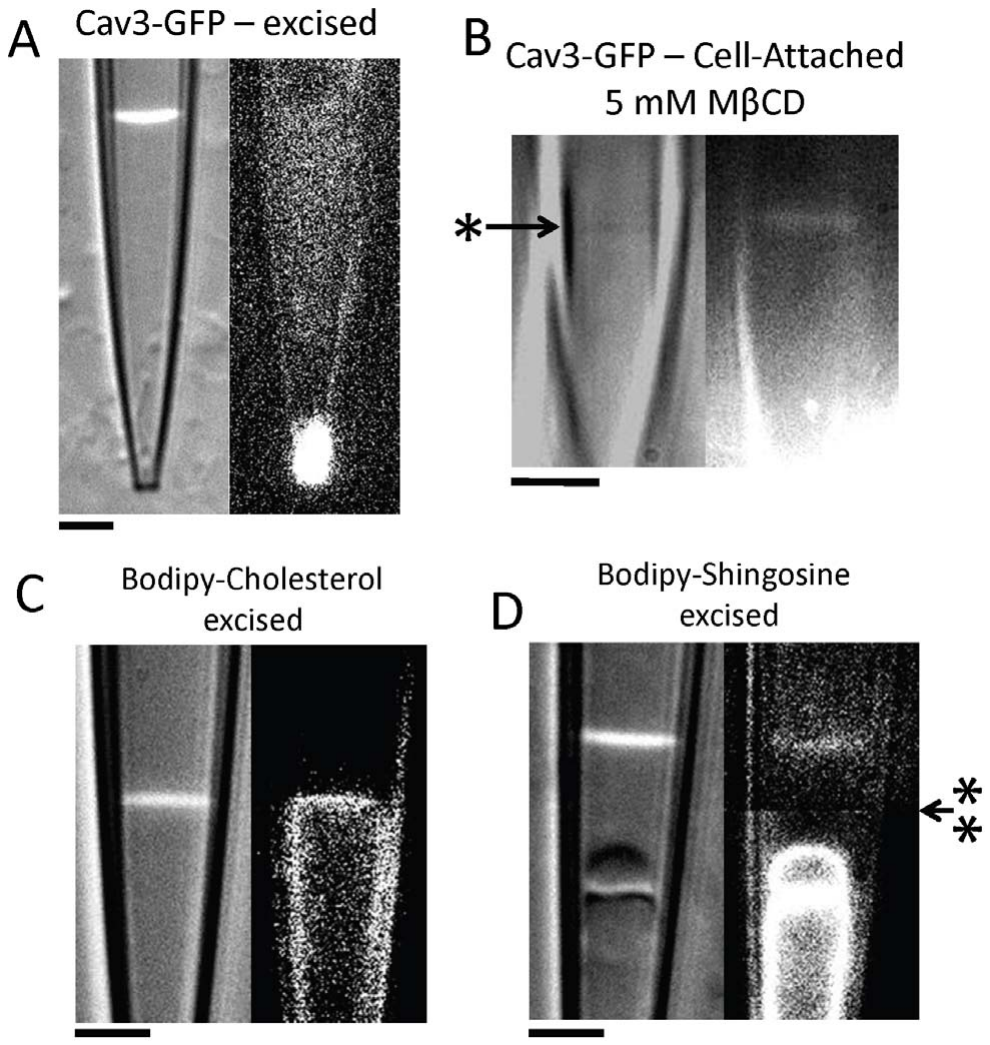
Localization of Cav3 and other membrane components in the patch. Representative DIC and fluorescence images of cell-attached and excised patches containing different fluorescently tagged molecules shows that, while Cav3 is normally excluded from the patch, other components of caveolae are present. (A) Shows an excised patch sealed at the tip with the electrode bent against the coverglass to make the patch dome parallel to the optical axis for better resolution of fluorescent protein localization. Weak fluorescence is uniformly distributed throughout the patch volume suggesting the fluorophore is not associated with the membrane. But bright fluorescence at the tip demonstrates segregation of Cav3 from the seal. (B) Shows a patch formed from a cell treated 1 hr with 5 mM MβCD. The difference in refractive index between the patch and the saline is significantly reduced due to the loss of cytoskeletal protein associations making the patch difficult to observe (the position of dome indicated by *). After this treatment, Cav3-GFP fluorescence is now clearly present in the patch dome but not in the seal region. Strong fluorescence from the cell produces higher background intensity reducing resolution of the fluorescence material in the lower half of the image closer to the cell. (C) An excised patch formed from a myotube stained with BODIPY labeled cholesterol. Cholesterol shows brighter fluorescence along the patch seal and dome clearly indicating membrane association. (D) BODIPY labeled sphingosine (Lactose-ceramide) also stains the dome and internal vesicles, but is excluded from the seal. Dome fluorescence was ∼3 times dimmer than the fluorescence associated with the internal vesicles suggesting some segregation of components occurs during patch formation. [The top half of the image was background subtracted (arrow with ** showing the spilt point) to remove fluorescent material on the coverslip.].

Cav3-GFP fluorescence in cell-attached (C/A) patches was difficult to accurately quantitate due to scattered fluorescent light from the much brighter adjacent cell and the angle of the pipette approach. Generally, a weak and diffuse fluorescence was observed throughout the patch volume. Only rarely (2 out of 28 patches) did we observe punctae that might represent caveolae under the dome. To reduce the background fluorescence, we excised patches and moved the tip away from the cells. We then pressed the tip against a clear portion of the coverslip bending it so that the patch dome became orthogonal to the optical axis (Fig. 6A). Even in this improved viewing mode, we only saw a weak diffuse fluorescence throughout the patch volume but intense fluorescence was observed at the pipette tip. In contrast, more than 50% of patches from myotubes expressing other GFP labeled proteins (TRPV2, TRPV4, TRPC6 and TREK) showed clear fluorescence in the patch dome but not in the seal region (see TRPC6 and TREK in [35]). Cav3-GFP had strong association with unstressed membranes since in 6 out of 28 patches, bright fluorescent labeled vesicles were present beneath the dome possibly derived from deeper reticulate structures sucked into the tip during seal formation (Fig. S8).

In contrast to untreated cells, patches formed from cells treated with MβCD showed fluorescence associated with the patch dome (3 out of 4 patches) though there did not appear to be any fluorescence in the seal region (Fig. 6B). Thus it appears that the normal structure of caveolae domains inhibits extraction of Cav3-GFP into the membrane that forms the patch, but dispersion of these domains with MβCD removes this constraint. In addition, the DIC image of MβCD treated patches had much lower contrast indicating a reduced difference in refractive index between the membrane and the pipette saline. This likely represents reduced cytoskeletal association with the dome.

Since Cav3 does not readily contribute to the patch formation, we decided to examine other components of the caveolae. Cholesterol and sphingolipids are major membrane components of caveolae microdomains. Patches from cells pretreated with BODIPY labeled cholesterol and the sphingolipid Lac-ceramide showed that cholesterol was present in both the patch dome and seal, while Lac-ceramide was only present in the dome. The Lac-ceramide distribution is similar to other fluorescent channel proteins [35] that are present in the patch dome but excluded from the seal. This result is consistent with the idea that membrane components that protrude far from the bilayer surface, such as membrane proteins and lipids with large carbohydrate head groups, are excluded from the seal, while molecules that can dissolve in the hydrophobic membrane interior (like FM1-43, [53]) can freely traverse the dome and seal.

### Whole Cell Indentation Induced Currents

**Figure 7:**
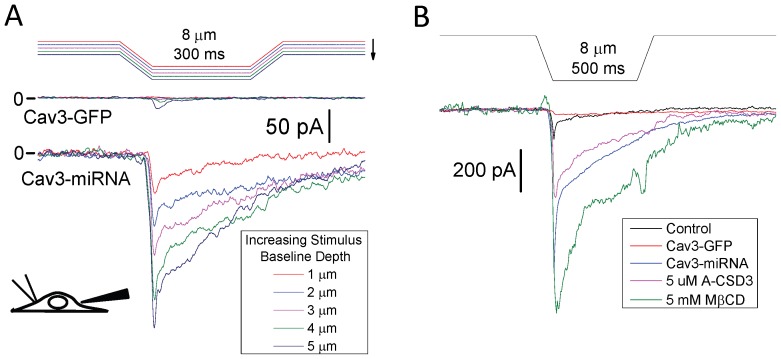
Indenting myotube sarcolemma shows that caveolae disruption affects whole cell MSC currents. Myotubes were whole cell voltage clamped at −60 mV membrane potential and indentation stimuli were applied ∼20 µm away from the patch pipette with a blunt glass probe (illustrated in the inset). Stimuli were trapezoidal waveforms of 8 µm depth and plateau durations of 300 and 500 ms (A and B respectively). (A) Stimulus intensity was increased by lowering the probe in 1 µm increments from the starting position. The currents induced from a single indentation of Cav3-miRNA transfected cells showed a response that increased nearly linearly with depth. The same indentations performed on a Cav3-GFP expressing cell showed significantly smaller currents. Similar to the ensemble patch currents, multiple stimuli were applied near the rupture depth (rupture depth determined after current acquired recordings) and averaged. Averaged currents from each cell were combined to produce an ensemble current for each condition (B). Cav3-miRNA expressing cells produced significantly larger currents (280 pA, n = 9) than control cells (30 pA, n = 7). Cav3-GFP overexpressing cells behaved like controls (20 pA, n = 7). Treatment with 5 µM A-CSD3 (10–20 min) and 5 mM MβCD (60 min) caused a significant increase in current (200 pA, n = 6 and 530 pA, n = 3 respectively). See Fig. S4C for statistical analysis.

We have shown here, and in previous studies [35], that the patch is a mechanically and chemically different environment compared to the host surface. To confirm the patch findings separate from the glass/membrane interactions of the patch, we measured whole cell currents from myotubes while indenting them with a blunt probe (Fig. 7) [53]. We used trapezoidal 8 µm indentations applied repetitively while the starting position was lowered in 1 µm increments (Fig. 7A). The starting point was when the steady state portion of the stimulus caused a visible indentation of the cell. The assay showed a nearly linear current response with indentation depth. Unlike the patch, these currents decayed during the stimulus which may be a combination of channel inactivation and mechanical adaptation [53]. As indentation depth increased, the cell would eventually rupture. Currents associated with rupture were easily distinguished from MSC currents since they showed a sudden increase in current magnitude (>10 times the current from the previous step), and resealed over tens of seconds. Because the myotubes were of variable thickness, we indented multiple times at each depth until the response showed signs of rupture. We created ensemble averages from the largest responses without signs of rupture for each condition (Fig. 7B). As with the patch assay, indentations of Cav3-GFP expressing cells produced relatively small currents that were not significantly different from control myotubes (Fig. S6D). Cells expressing Cav3-miRNA and treated with MβCD or A-CSD3 peptide showed significantly larger indentation currents than the controls, having actual magnitudes comparable to those observed in the patch. However, unlike the non-inactivating patch currents, these currents show a strong phasic behavior. The differences in responses between the two assays is likely due to two multiple processes. First, the disruption and reformation of the cortical cytoskeleton in patch membrane may cause loss of the labile inactivation property observed in most MSCs in other cells and in outside-out patches from myotubes [2], [36], [61], [62]. Second, while the force of the pressure stimulus in the pipette is continuous, the force due to probe indentation likely relaxes at the plateau phase of the stimulus. The stress/strain distribution around the probe is not known in detail so quantitive comparisons are not dependable. Finally, the complement of channel types present in the patch dome compared to those around the probe tip may differ producing the phasic behavior. However, these data qualitatively agree with the patch data and show that, even though significant biophysical differences exist between the two assays, Cav3 depletion or caveolae disruption increases MSC activity.

## Discussion

### Caveolae/Cav3 Couple MSC Function with Cytoskeletal Tension Control

**Figure 8:**
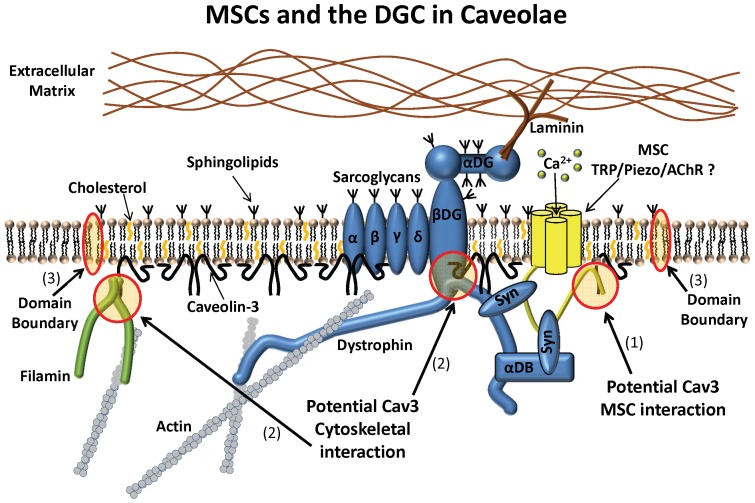
Model of the dystroglycan complex and Cav3 interactions that may affect MSCs. Three potential sites of MSC modulation are highlighted: (1) Cav3 may interact directly with channels; (2) The Cav3 WW domain could compete with β-dystroglycan at the dystrophin binding site reducing cytoskeletal linkages. The Cav3 scaffolding domain interacts with other cytoskeletal crosslinking proteins like filamin. Changing Cav3 abundance or using inhibitors directed at specific binding partners may shift the equilibrium of these associations and alter membrane mechanics and MSC activity; (3) The transition zone between the cholesterol/shingolipid rich caveolae domains and the bulk membrane may prevent caveolae components from freely diffusing. Changes to other components of the DGC complex could occur by any of the means used to affect Cav3 interactions which could indirectly affect MSCs through membrane tension buffering (ex. dystrobrevins) or sequestration of MSCs (ex. syntrophins). Protein abbreviations: **Syn** – syntrophin, **αDB** – α-dystrobrevin, **βDG** – β-dystroglycan, **αDG** – α-dystroglycan.

An important means of regulating signaling complexes contained within caveolae involves controlling the availability of receptors to the stimulus and receptor interactions with effectors and modulating factors [3], [21], [63], [64]. Through various acute and chronic means of affecting caveolae structure, we have shown that caveolae are involved in regulating MSC currents in muscle cells. One way appears to be controlling the local stress. In the patch assay, increased disruption of caveolae or Cav3 interactions, or reduction in Cav3 protein was correlated with increased MSC currents and a reduction in the membrane relaxation rate. A similar current increase was observed in the indentation assay for the various methods of caveolin disruption, strengthening the argument that caveolae are involved in MSC regulation. As expected, these studies suggest multiple potential mechanisms of MSC modulation in caveolae. Some potential mechanisms are highlighted in Figure 8, including (1) direct interaction of Cav3 with the channels, (2) Cav3 modulation of cytoskeletal association with the membrane affecting local stress, and (3) sequestering MSCs (and other components) in caveolae microdomains, which are discussed below.

#### (1) Direct Cav3 interaction with channel

When a low concentration of A-CSD3 was first applied to cells there was an increase in MSC activity with no corresponding change in ΔCp. This would suggest that a direct association between MSCs and Cav3 exists since patch mechanical changes did not occur until later. Caveolin associates with many channel types through its scaffolding domain [32] and creating signaling complexes with multiple adaptor proteins [23]. TRP channels are included in this list of interactions and have been implicated in mechanosensitivity and the elevated Ca^2+^ levels in dystrophy. TRPC1 and TRPC4 knockdown in myofibers reduces the elevated Ca^2+^ leak in dystrophic *mdx* fibers [65], and A-CSD1 peptide disrupts receptor induced Ca^2+^ influx through TRPC1 channels in endothelial cells [59]. In myotubes, ROS activation of TRPC1 and the associated Ca^2+^ influx were linked to Cav3 coexpression [3], though TRPC1 does not appear to comprise the MSCs in myofibers [66].

Although there are no reports of direct Cav3 interactions with TRPV2 or TRPV4, both channels are expressed in myotubes and myofibers and have been linked to MSC activity and elevated Ca^2+^ in normal and *mdx* myofibers [6]–[8]. A new class of cationic-MSCs channels called the Piezo family [10], show rapid inactivation similar to the endogenous MSCs in myotube outside-out patches [2] and both are inhibited by GsMTx4 [38]. While the level of RNA expression for these channels is comparatively low in muscle tissue, this is not a sensitive indicator of phenotypic expression. It has recently been suggested that nicotinic acetylcholine receptors endogenously expressed in C2C12 myotubes are mechanosensitive in patches, although the effects seem to be a change in the number of mechanosensitive channels rather than changing the gating of individulal channels [9]. The currents are blocked by GsMTx4 and the channels are clustered in caveolae at the neuromuscular junction [64].

The single channel MSC currents we observed in these studies have multiple conductance levels and their sensitivity to pressure varies (although this can be a result of identical channels being in different mechanical domains) so that they likely represent a variety of channel types. The control cells and most treatment groups (Cav3-GFP, MβCD and A-CSD3) showed a preponderance of channels with conductances in the range of 20–40 pS. However, patches from Cav3-miRNA treated cells showed a strong shift toward higher conductance channels. This may be a change in the developmental expression pattern in the absence of Cav-3 as opposed to a specific channel interaction since both MβCD and A-CSD3 which would disrupt specific channel interactions did not show a shift to larger conductances. This emphasizes that these methods of affecting caveolae are not equivalent and that increased current in patches from Cav3-miRNA expressing cells may result from changes in both mechanical and channel properties.

There have been a number of reports describing the primary conductance levels, and the differences in kinetic properties and sensitivities to pressure stimuli for MSCs observed in rodent myotubes and myofibers [1], [2], [6], [66]. Most find conductances in the range of 10–50 pS. Some of these differences may result from the different developmental stages studied [1], while others may arise from differences in the ionic composition of the recording solutions. Another difference is whether brief pressure steps or long duration pressure stimuli are applied since different channels may dominate during transient stimuli vs those at steady state. We are currently investigating the source of the channels which are affected by Cav3.

#### (2) Cav3 modulation of cytoskeletal association with the sarcolemma

It is clear that the normal forces in the cortical cytoskeleton can overcome the adhesion energy of the gigaseal. Seal tension alone would flatten a dome bilayer unaffected by other forces. But images of patches show that the dome can take on highly irregular geometries when no pressure is applied [35]. The geometry of the patch has multiple local folds in rapidly remodeling cells like HEK cells, whereas myotubes are more mechanically robust and mainly show differences in uniform dome curvature. When tension is applied to patches where the cytoskeleton has been disrupted by either chemical (Latrunculin/cytochalasin [2], [36]) or mechanical means (excised patches [35], [36]), the dome becomes flat and relaxation rates become more rapid. However, it is not easy to predict how changes to specific cytoskeletal elements will affect the patch mechanics as when dystrophin is missing the patch relaxation rate actually increases.

The capacitance data clearly shows that treatments that disrupt caveolae structure also modify membrane mechanics. The trend emerged showing that as patch relaxation rates decreased (less viscous), MSC currents increase. Cytoplasmic viscosity probably reflects primarily the time to break non-covalent bonds of crosslinkers.

Cav3 is a key regulator for cortical cytoskeletal interactions with the sarcolemma [67], [68]. The data from Cav3 overexpression, Cav3 knockdown and cholesterol depletion led us to assume that Cav3 control of MSCs is a simple function of cytoskeletal/membrane interactions. Cholesterol depletion, that is known to reduce association of cytoskeletal anchoring proteins with the membrane [55], [56], produced the most significant change in relaxation. Imaging of patches from MβCD treated cells clearly showed that the refractive index of the dome membrane has decreased suggesting there is a loss of associated cytoskeletal protiens.

Treatment of cells with 10–20 µM A-CSD3 seems to confirm the function of Cav3 as a cytoskeletal modifier, since this treatment rapidly disrupts the cytoskeleton leading to an enormous release of membrane. However, low peptide concentrations exposed a more complex function by initially increasing MSC currents with no corresponding change in ΔCp (i.e. patch mechanics). Longer treatments, which presumably would increase caveolae disruption, caused membrane stiffening and decreased channel activity. We have two alternative explanations for the latency of cortical stiffening. The first is that the low level Ca^2+^ leak may signal cytoskeletal changes to reinforce the cell cortex. The second is differences in the affinity of Cav3 for various binding partners found in caveolae. We did not measure the rate of A-CSD3 accumulation within the cell, though it is possible that the initial MSC current increase is caused by disruption of a relatively weak interaction since at early times the internal concentration is low. Based on the study of A-CSD1 internalization by Kwiatek [59], the concentration is likely only in the low nanomolar range at early times. Cav3 binding partners are numerous, but some stand out as critical to control sarcolemma tension. For example, in the dystroglycan complex, Cav3 competes with dystrophin for the β-dystroglycan binding site [69] and may regulate the level of dystrophin interaction. Cav3 also directly interacts with other cortical reinforcing components such as filamin that crosslinks f-actin to integrins [67].

The mechanoprotective effect of caveolae on MSCs has consequences for understanding muscular dystrophy. In dystrophic myofibers (lacking dystrophin) there is increased Cav3 expression [70] and an increased number of caveolae [71] suggesting that this may be an adaptation of cells with a mechanically compromised sarcolemma. In a previous report we showed that *mdx* dystrophic myotubes have slower patch relaxation rates than normal which may be a function of increased Cav3 expression. Also, *mdx* patches had similar MSC activation levels as normal cells. But resting currents were elevated probably due to greater patch compliance [2]. This increased compliance transfers more energy to the bilayer where the MSCs respond. Thus, increasing Cav3 expression in the absence of dystrophin may increase other membrane-cytoskeletal associations, but this adaptation is incapable of providing the same mechanoprotection as a functional DGC.

#### (3) MSC sequestration in caveolae lipid domains

Patches are formed from membrane blebs generated by suction in the pipette [35]. The protein and lipid composition of blebs is different from the undisturbed sarcolemma [39], [72]. The bright fluorescence of Cav3-GFP at the pipette tip and its absence from the patch dome and seal of untreated cells suggests that Cav3 is excluded from the membrane bleb that forms the patch. Importantly, this segregation may extend to other resident components of caveolae. The line tension present at the transition zone between caveolae domains and the surrounding membrane probably poses an energy barrier to the free diffusion of proteins into and out of the domain [31]. Alternatively, Cav3 interactions with actin-membrane crosslinkers may form “corrals” inhibiting release of components from these domains during patch formation [63], [73]. Interestingly, besides control patches (71% MSC positive patches) all conditions tested (Cav3-GFP, Cav3-miRNA, MβCD, and A-CSD3) showed >80% levels of MSC positive patches. Thus it would appear that MSCs sequestration is not a significant factor. However, the average currents do not distinguish between greater numbers of channels and greater open probability. We were unable to make this distinction because the data from a single patch in most cases is refractory to these analyses due to the record noise from multiple channel openings with different main conductance and subconductance states. In addition, even if the channel number analysis were possible it would not distinguish between mechanoprotection by reduced channel presence in the patch or reduced activatable channels.

In the indentation assay, MSC currents in normal cells were rarely elicited by gentle, shallow indenting of the myotubes. Indentations of 5–10 µm into cells that are 10–15 µm thick were normally required to induce currents. Similar experiments on adult ventricular cardiomyocytes required large indentations for minutes before the cytoskeleton broke exposing MSC activity [74]. Together these data suggest that caveolae may create corrals shielding MSCs from minor stresses in normal cells. Significant mechanical disturbances, such as those occurring during patch formation or large indentations disrupt the domains and expose the MSCs to membrane stress. Mutations that weaken the cortex and cause muscular dystrophy may lower the mechanical threshold for release of MSCs to less mechanically buffered bilayer regions.

### Patch Composition

Though caveolae are abundant in the sarcolemma and may deform to buffer membrane stress by providing expandable domains [19], [20], they likely do not perform this function in the patch dome since fluorescence imaging shows Cav3 is not detectable in patches from untreated cells. Differences in Cav3 levels more likely act by affecting the cytoskeletal interactions with the dome membrane of the patch. So how can Cav3 levels affect the patch cytoskeletal associations if it isn’t in the seal or dome? One possibility is that Cav3 levels alter the relative abundance of cortical cytoskeletal components and cholesterol that make it to the dome during seal formation [23]. Since the bleb that forms the patch is initially devoid of cytoskeletal associations and has different composition from the surrounding undisturbed sarcolemma [39], [75], most cytoskeletal interactions in the dome occur after seal formation. After seal formation diffusible cytoplasmic factors can associated with the dome, but membrane components outside of the pipette will be restricted from entering the patch by the gigaseal barrier. Sphigolipids and all channel proteins we have observed thus far are excluded from the seal but do appear in the dome at varying concentrations depending on their ability to partition into the bleb before the seal forms.

Cholesterol depletion caused Cav3 to become uniformly distributed over the sarcolemma and to appear in the patch dome. Apparently, when not contained within caveolae, Cav3 is no longer restricted from entering the patch. Excised patches from MβCD treated cells were fragile and did not last long enough after patch excision and to be moved to a clear area of the coverslip for viewing. However, in cell-attached mode, it appears that Cav3 does not associate with the seal region or at least is not as bright as the dome. This was surprising since Cav3 is thought to interact only with the inner leaflet. However, its exclusion from the seal region would suggest either the ordering or composition of the monolayer in contact with the glass affects the properties of the inner leaflet making it unfavorable for Cav3 occupancy, or Cav3 is associated with membrane proteins that do not enter the seal.

### Patch Suction Compared to Whole Cell Indentation

MSC patch currents increased during the stimulus while those evoked from the indentation stimulus were transient in nature. This decay in the indentation current is likely due to a combination of channel inactivation, channel type differences and viscous adaptation of the stimulus in the pressing assay [52]. The channels activated in the indentation assay may be different than those activated in the patch or may remain associated with membrane and protein components that confer the inactivating properties on the channels. Also, in the patch the stress in the dome is mostly homogeneously distributed due to the relatively rigid boundary where the membrane adheres to the glass wall and the mixing of components during formation. In contrast, the force from the indentation stress is distributed in a gradient manner away from the probe and the flow of membrane to relieve stress at the probe contact region is less impeded so the stress may actually peak and then decline during the stimulus depending on the viscosity of the system. We cannot monitor the stress during indentations as we did in the patch, but it allows testing MSC currents in a more native setting than the patch assay and showed the same basic channel response as the patch.

### Summary

The main findings of these studies are that conditions that tend to disrupt caveolae lead to greater MSC currents, while stabilizing conditions had a mechanoprotective effect. This mechanoprotection is complicated due to the many interactions involving Cav3. However, a common thread appears to be through caveolin’s role in organizing the cortical cytoskeleton near the channels and reducing stress in the bilayer where the channels are located. This is based on the observations that disruption or depletion of caveolae leads to a significant increase in MSC activation in both the patch and indentation assays which is accompanied by an increase in the rate of membrane relaxation in the patch assay. In addition, while increased Cav3 expression did not have a significant effect on either assay, all measures were trending toward lower MSC activity and increased membrane stiffening suggesting the system is saturated. Specific interruption of Cav3 interactions with A-CSD3 shows that the mechanoprotective effect is complex with multiple channel and cytoskeletal interactions that vary in affinities for Cav3.

While the physiological function of MSCs in myotube is uncertain, it is likely they play a significant role as “safety alarms” that inform the cell of excess bilayer stress and signal cytoskeletal reinforcement to prevent lysis. A compromised cytoskeletal is common to most forms of dystrophy leading to loss of mechanoprotection and hyperactivity of MSCs making these channels an important target for new drug therapies. The unique physical properties of caveolae membranes might lend themselves to the development of new compounds to target the localized channels. Future studies need to elucidate the specific interactions between caveolae and MSCs and determine if MSCs actually reside in these membranes.

## Supporting Information

Figure S1 **Transfection effects on myotube development.** Representative images of myotube cultures. Control (untransfected) myocyte cultures produced few myotubes. Fugene 6 reagent alone increases myotube development. Fugene 6+ DNA (Cav3-GFP or Cav3-miRNA) strongly potentiated myotube development.
(PDF)

Figure S2 **Age related maturation of myotube cytoskeleton.** DIC and fluorescence images of myotubes expressing fluorescently tagged actinin shows significant differences in the sarcomeric structure between 7 and 14 days. These studies were performed on myotubes ranging in age from 10–20 days post plating.
(PDF)

Figure S3 **Additional examples of single channel MSC recordings and average currents.** (A–C) Show currents as in Figure 2 for three additional patches with different channel conductances and gating kinetics. Each set of recordings shows four representative single channel current recordings at −120 mV membrane potential and conductance recordings at 0 mV membrane potential. Beneath each recording is a red trace showing the average current from 5–15 presure steps. All records were from −70 mmHg stimuli. The 3 recordings show examples of MSC currents from different conductance groups; (A) ∼25 pS channels, (B) ∼50 pS channels, and (C) ∼100 pS channels. The kinetics of the largest conductance channels (C) were very distinct from the lower conductance ones, having shorter open times. (C) Is an example of a patch that likely contains both a low and high conductance MSCs. For example see traces 2 and 3 in the conductance panel indicated by asterisk.
(PDF)

Figure S4 **Increased temperature increases MSC activity in 14 day myotubes.** (A) Ensemble average MSC currents and ΔCp at 21°C and 37°C in myotubes that were ∼14 day old (−110 mV membrane potential). Control MSC currents came from untransfected or GFP control vector transfected cells (n = 20 @ 21°C, n = 28 @ 37°C). Increasing the temperature from 21°C to 37°C increased the average patch currents with a Q_10_ of ∼1.6 [where Q_10_ = (I_2_/I_1_)^10/(T2−T1)^] consistent with the expected ionic conductance changes for a 16°C temperature difference. (B) Shows the ensemble averaged MSC currents measured during the stimulus at different pressures and temperatures. (C) The measured average magnitude of the capacitance changes for the two cell types showed no significant difference at either temperature or pressure. However, the estimated fitting data shows that the relaxation rate at 37°C was ∼200 ms longer and the steady state ΔCp was ∼40% greater than at 21°C (D). A portion of the difference in ΔCp magnitude may arise from the more rapid patch creep at 37°C so that the patch dome area was ∼25% greater than at 21°C at all pressures (2.96±0.07 µm diameter at 21°C, 3.41±0.06 µm diameter at 37°C). The differences in mechanical relaxation rates may arise from temperature dependent changes in the elastic properties of the cytoskeleton.
(PDF)

Figure S5 **Time dependent patch mechanical properties.** (A) ΔCp was measured on patches stimulated first with a 500 ms suction steps and followed by a 2500 ms steps to −60 mmHg, to create ensemble averages (n = 21). Patches with a large range of time constants were chosen for comparison. We lowered the suction from the standard −70 mmHg to −60 mmHg because many of the patches ruptured after only a few 2500 ms steps or showed random abrupt capacitance deflections possibly related to significant changes in the patch structure. While the 500 ms rising phase has kinetics similar to the 2500 ms curve, plotting the individual rates from 500 ms against those at 2500 ms (B) shows that the rates from 500 ms stimuli were ∼20% shorter as determined by a linear regression fit to the data. The estimated steady state ΔCp from the 500 ms steps was 24.1±2.7 fF. This was ∼15% lower than the steady state ΔCp (∼28 fF) measured from the 2500 ms data curve. However, the 2500 ms curve never reached a true steady state due to patch creep. This is shown by the significant displacement of the baseline indicated by the arrow at the end of the relaxation descending phase (A) when the pressure returns to 0 mmHg. The more stable data of the 500 ms pressure steps was selected for analysis.
(PDF)

Figure S6 **Statistical comparison of MSC currents under different conditions.** Ensemble averaged patch and indentation induced currents are shown for control myotubes expressing different exogenous proteins or control myotubes under different conditions. (Panels A, B and D) show MSC currents during the pressure stimulus. (C) Shows the average patch current during the 0 mmHg period 0.5 sec prior to the start of the pressure steps for reference. (**) Denotes statistical differences at the 5% level of significance. (*)Denotes statistical differences at the 10% level of significance. ***Capacitance:*** Statistical analyses of ΔCp with −70 mmHg pressure steps. The magnitude of ΔCp for Cav3-miRNA expression and 5 mM MβCD treated cells were significantly greater than for the control patches (5% level of significance with a probability of 0.0174 and 6.14×10^−6^ respectively). The magnitude of ΔCp for A-CSD3 treated cells after 30 min was significantly less than the control patches (5% level of significance with a probability of 0.0176). The magnitude of the ΔCp for Cav3-GFP expression and A-CSD3 treated cells at 10 min were not significantly different from the controls indicating no effect on membrane elasticity.
(PDF)

Figure S7 **Cav3 associates strongly with unstressed internal membranes.** Shows DIC and fluorescent images of an excised patch from a cell expressing Cav3-GFP pressed against coverslip. Cav3-GFP laden vesicles fill the space beneath the dome suggesting Cav3-GFP can readily associate with unstressed membranes.
(PDF)

Figure S8 **A-CSD3 effect is sensitive to external Ca^2+^ but not VACC inhibitors.** Fluo 4 loaded myotubes were monitored after application of 10 µM A-CSD3 to the bath in the absence of external Ca^2+^ or in the presence of a cocktail of voltage activated calcium channel (VACC) inhibitors (50 µM Nifedipine, 50 µM Verapamil and 50 µM Diltiazem). VACC inhibitors had no effect on the A-CSD3 induced Ca^2+^ influx. In the absence of external Ca^2+^, the response induced by A-CSD3 was delayed and reduced in amplitude.
(PDF)

Movie S1 **Immunofluorescence shows normal Cav3 distribution in myotubes.** Representative through focus z-stacks of a myotube showing the distribution of Cav3. Fluorescence and DIC 0.3 µm sections through a Cav3 immunofluorescence labeled normal myotube showing the endogenous distribution. Cav3 has a punctate distribution on the cell surface and a reticulate internal structure.
(AVI)

Movie S2 **Cav3-GFP fluorescence shows distribution similar to endogenous Cav3.** Representative through focus z-stacks of a myotube showing the distribution of Cav3-GFP fusion protein. Similar to the endogenous Cav3 distribution in Movie S1, 0.5 µm optical sections through a Cav3-GFP expressing myotube shows expression at the myotube surface and internally.
(AVI)

Movie S3 **Cav3-GFP distribution after treatment with 5 mM MβCD.** Representative through focus z-stacks of a myotube showing the distribution of Cav3-GFP fusion protein. Acquiring optical sections of 0.5 µm thickness through a Cav3-GFP expressing myotube after treatment with 5 mM MβCD shows Cav3-GFP becomes diffuse on the sarcolemma and multiple vesicles form internally.
(AVI)

Movie S4 **A-CSD3 peptide causes Ca^2+^ influx associated with membrane detachment.** Myotube cultures were loaded with Fluo4-AM, and DIC and fluorescent images were acquired every 3–4 min at 37°C. Cells were treated with 17 µM A-CSD3 at 12–15 min (∼frame 4) after which they rapidly took up Ca^2+^, contracted and released large amounts of membrane. The area of membrane released from the cells sometimes formed spheres with a diameter close to the longitudinal length of the original cell. This reinforces the fact that the membrane comprising the sarcolemma and t-tubule system is well in excess of the observable surface area of a myofiber [1]. Total video time = 117 min.
(AVI)

Movie S5 **Cholesterol depletion causes some myotube disruption but slower than A-CSD3.** Treatment of Fluo4-AM loaded myotubes with 5 mM MβCD at ∼ frame 4 did not produce significant Ca^2+^ influx and only some cells were disrupted. Total video length = 156 min. 1. Pasek M, Brette F, Nelson A, Pearce C, Qaiser A, et al. (2008) Quantification of t-tubule area and protein distribution in rat cardiac ventricular myocytes. Prog Biophys Mol Biol 96: 244–257.
(AVI)
