# Supplementary figures and images for: Caveolae Regulation of Mechanosensitive Channel Function in Myotubes
Source: PLoS One. 2013 Aug 30;8(8):e72894. doi: 10.1371/journal.pone.0072894 (PMC3758351; doi:10.1371/journal.pone.0072894)

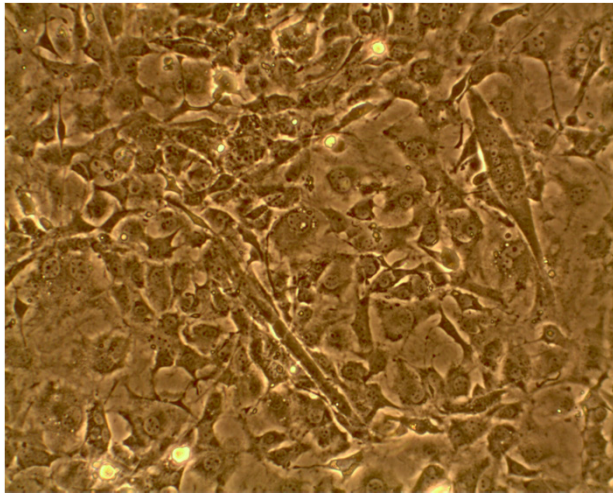

control

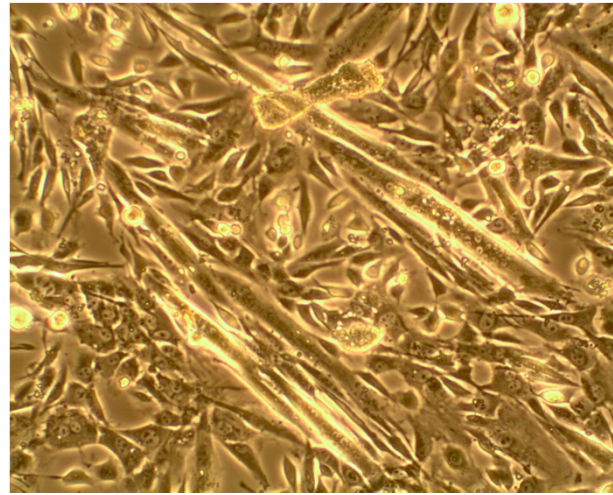

Fugene 6

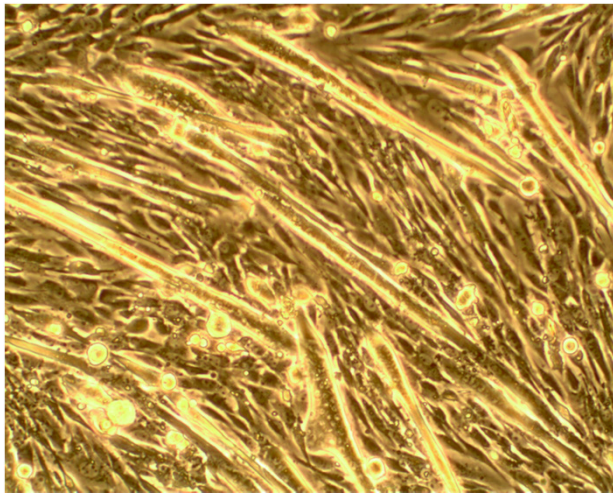

Cav3-GFP + Fugene 6

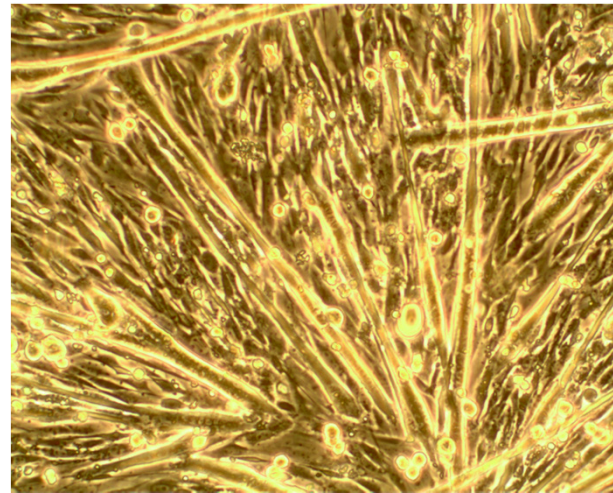

Cav3-miRNA + Fugene 6

Supporting  
Figure S1

Supplement: Figure S1 — Transfection effects on myotube development. Representative images of myotube cultures. Control (untransfected) myocyte cultures produced few myotubes. Fugene 6 reagent alone increases myotube development. Fugene 6+ DNA (Cav3-GFP or Cav3-miRNA) strongly potentiated myotube development. (PDF) [file pone.0072894.s001.pdf]

## Actinin Expression

7 Days

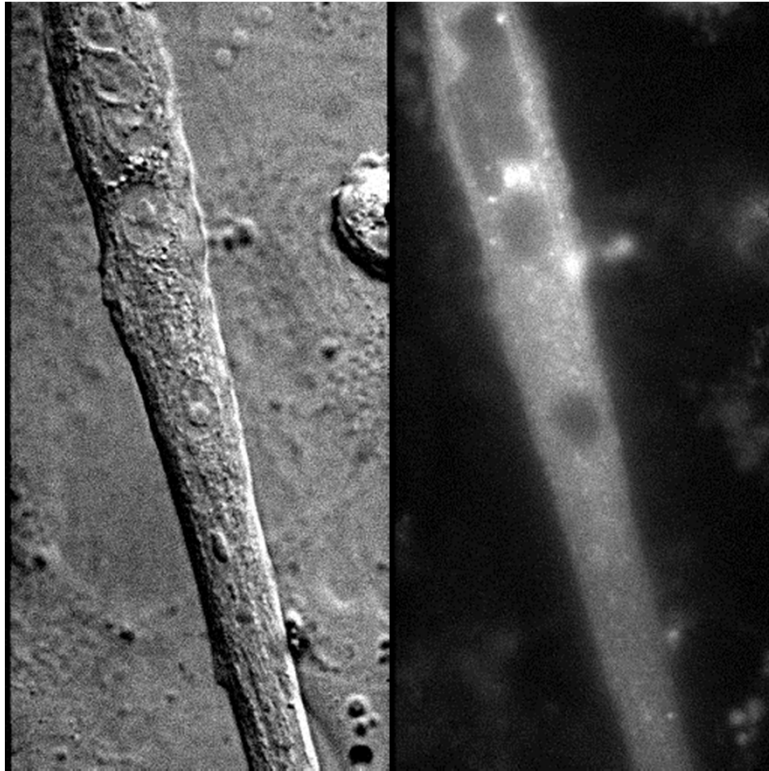

14 Days

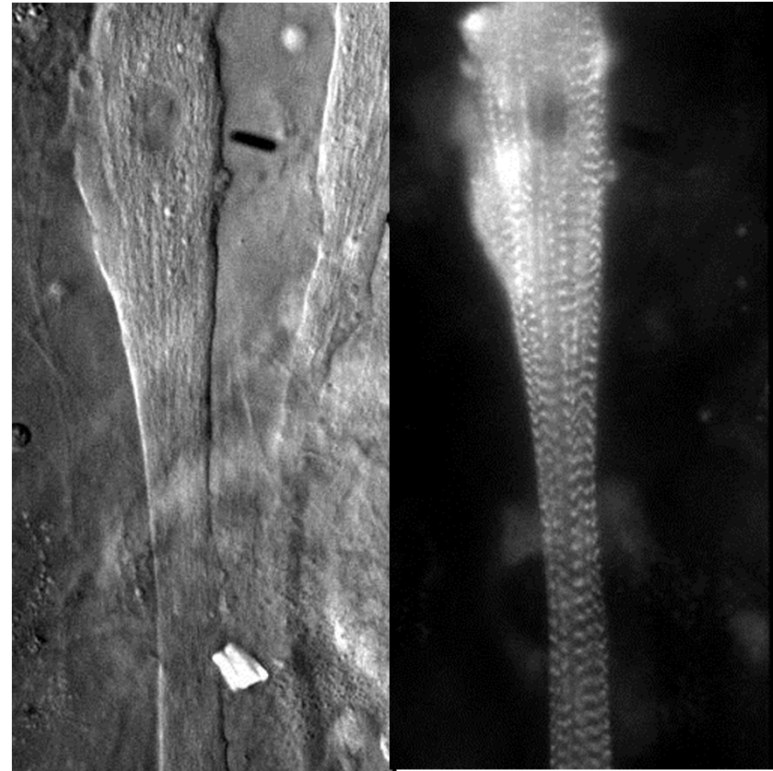

Supporting  
Figure S2

Supplement: Figure S2 — Age related maturation of myotube cytoskeleton. DIC and fluorescence images of myotubes expressing fluorescently tagged actinin shows significant differences in the sarcomeric structure between 7 and 14 days. These studies were performed on myotubes ranging in age from 10–20 days post plating. (PDF) [file pone.0072894.s002.pdf]

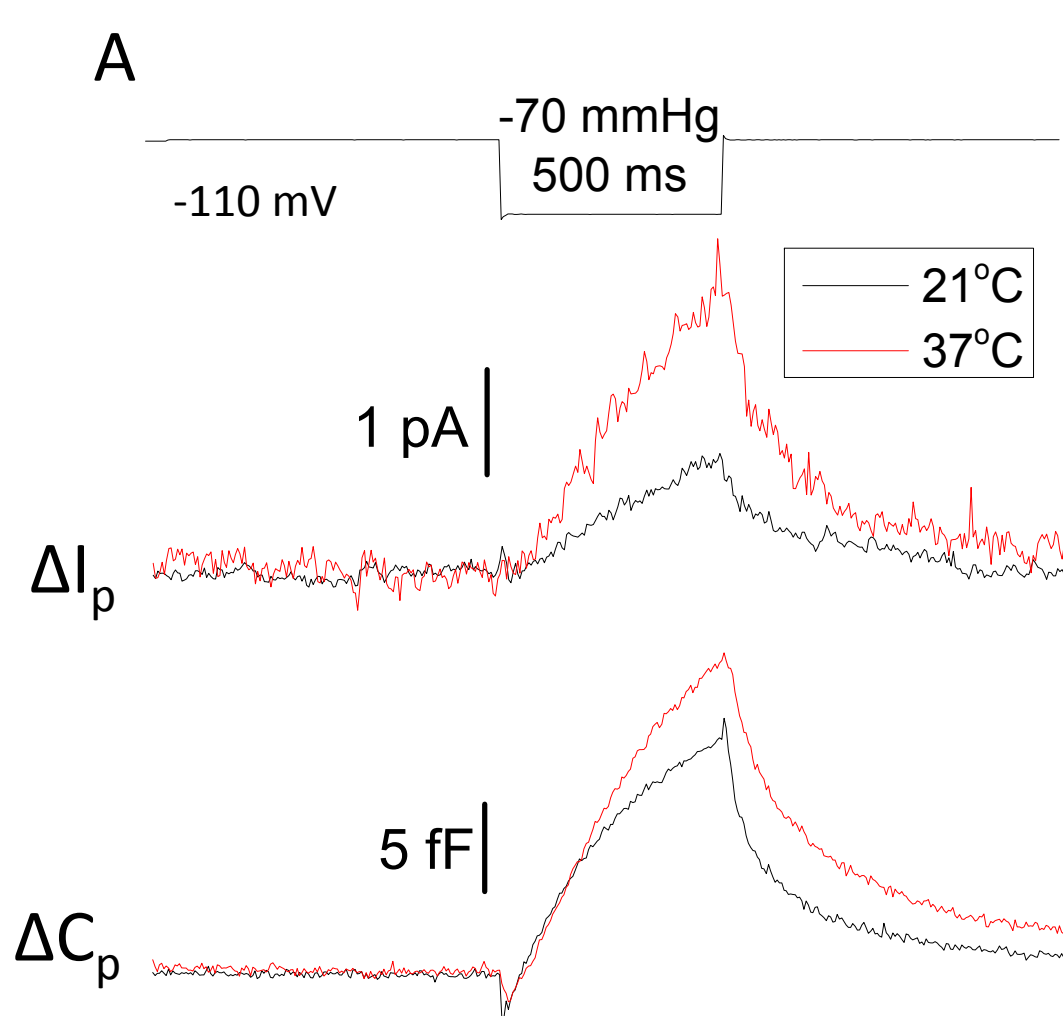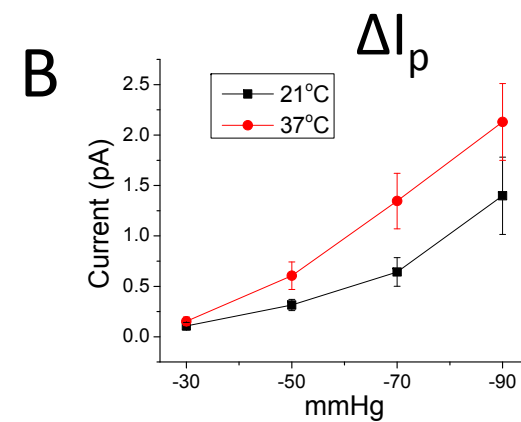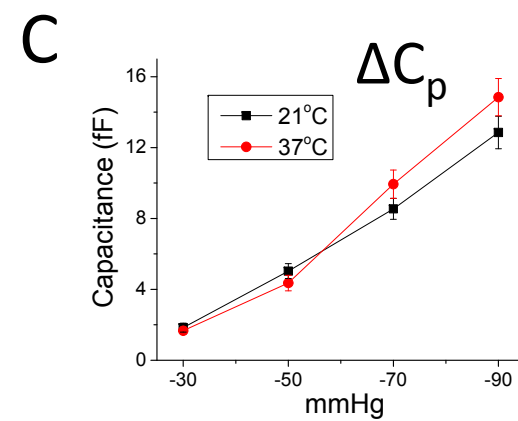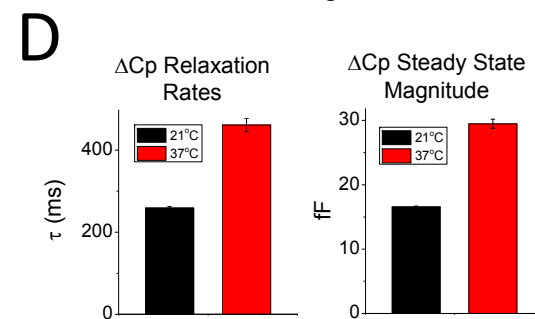

Supporting  
Figure S4

Supplement: Figure S4 — Increased temperature increases MSC activity in 14 day myotubes. (A) Ensemble average MSC currents and ΔCp at 21°C and 37°C in myotubes that were ∼14 day old (−110 mV membrane potential). Control MSC currents came from untransfected or GFP control vector transfected cells (n = 20 @ 21°C, n = 28 @ 37°C). Increasing the temperature from 21°C to 37°C increased the average patch currents with a Q10 of ∼1.6 [where Q10 = (I2/I1)10/(T2−T1)] consistent with the expected ionic conductance changes for a 16°C temperature difference. (B) Shows the ensemble averaged MSC currents measured during the stimulus at different pressures and temperatures. (C) The measured average magnitude of the capacitance changes for the two cell types showed no significant difference at either temperature or pressure. However, the estimated fitting data shows that the relaxation rate at 37°C was ∼200 ms longer and the steady state ΔCp was ∼40% greater than at 21°C (D). A portion of the difference in ΔCp magnitude may arise from the more rapid patch creep at 37°C so that the patch dome area was ∼25% greater than at 21°C at all pressures (2.96±0.07 µm diameter at 21°C, 3.41±0.06 µm diameter at 37°C). The differences in mechanical relaxation rates may arise from temperature dependent changes in the elastic properties of the cytoskeleton. (PDF) [file pone.0072894.s004.pdf]

Supporting  
Figure S5

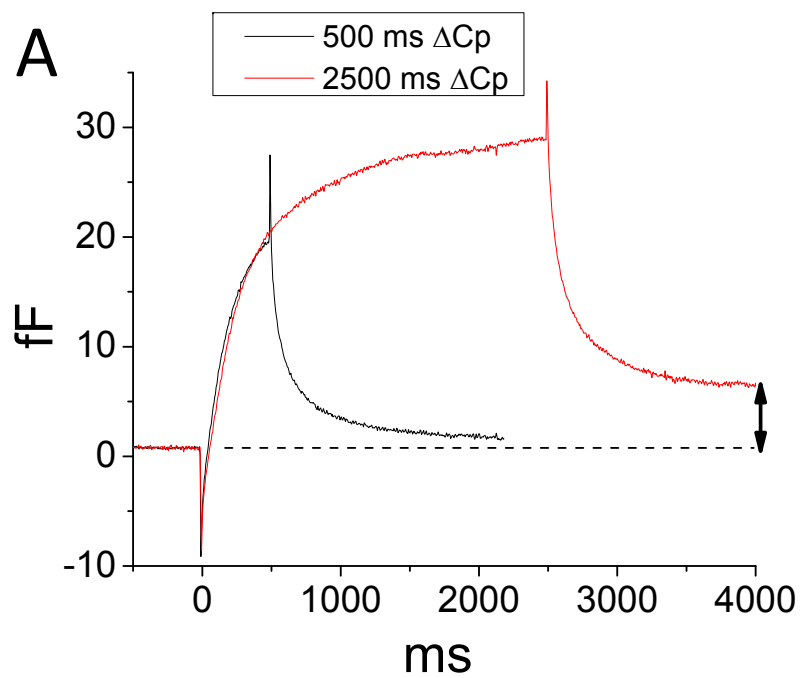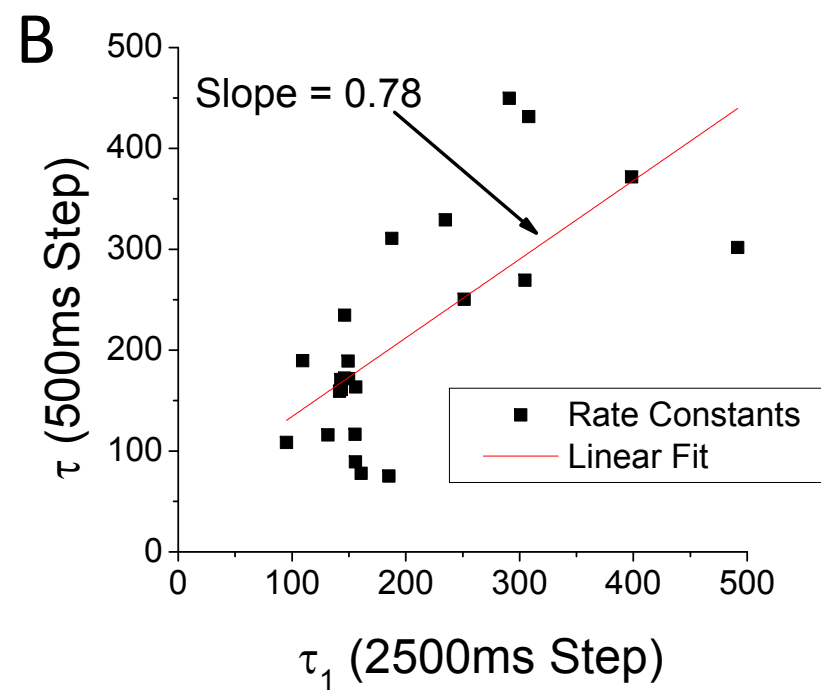

Supplement: Figure S5 — Time dependent patch mechanical properties. (A) ΔCp was measured on patches stimulated first with a 500 ms suction steps and followed by a 2500 ms steps to −60 mmHg, to create ensemble averages (n = 21). Patches with a large range of time constants were chosen for comparison. We lowered the suction from the standard −70 mmHg to −60 mmHg because many of the patches ruptured after only a few 2500 ms steps or showed random abrupt capacitance deflections possibly related to significant changes in the patch structure. While the 500 ms rising phase has kinetics similar to the 2500 ms curve, plotting the individual rates from 500 ms against those at 2500 ms (B) shows that the rates from 500 ms stimuli were ∼20% shorter as determined by a linear regression fit to the data. The estimated steady state ΔCp from the 500 ms steps was 24.1±2.7 fF. This was ∼15% lower than the steady state ΔCp (∼28 fF) measured from the 2500 ms data curve. However, the 2500 ms curve never reached a true steady state due to patch creep. This is shown by the significant displacement of the baseline indicated by the arrow at the end of the relaxation descending phase (A) when the pressure returns to 0 mmHg. The more stable data of the 500 ms pressure steps was selected for analysis. (PDF) [file pone.0072894.s005.pdf]

# Statistical Significance

## Supporting Figure S6

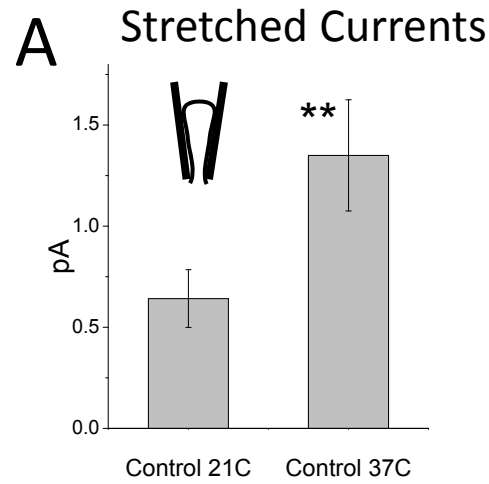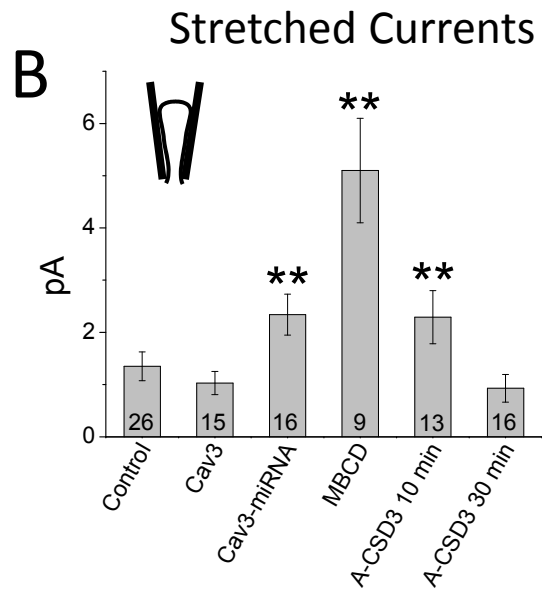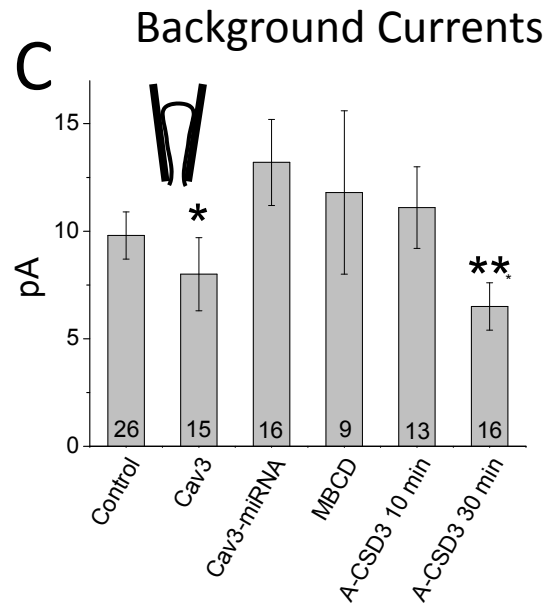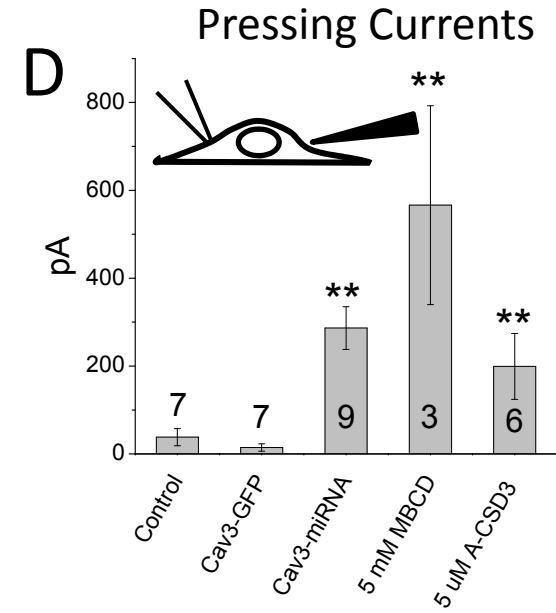

Supplement: Figure S6 — Statistical comparison of MSC currents under different conditions. Ensemble averaged patch and indentation induced currents are shown for control myotubes expressing different exogenous proteins or control myotubes under different conditions. (Panels A, B and D) show MSC currents during the pressure stimulus. (C) Shows the average patch current during the 0 mmHg period 0.5 sec prior to the start of the pressure steps for reference. (**) Denotes statistical differences at the 5% level of significance. (*)Denotes statistical differences at the 10% level of significance. Capacitance: Statistical analyses of ΔCp with −70 mmHg pressure steps. The magnitude of ΔCp for Cav3-miRNA expression and 5 mM MβCD treated cells were significantly greater than for the control patches (5% level of significance with a probability of 0.0174 and 6.14×10^−6^ respectively). The magnitude of ΔCp for A-CSD3 treated cells after 30 min was significantly less than the control patches (5% level of significance with a probability of 0.0176). The magnitude of the ΔCp for Cav3-GFP expression and A-CSD3 treated cells at 10 min were not significantly different from the controls indicating no effect on membrane elasticity. (PDF) [file pone.0072894.s006.pdf]

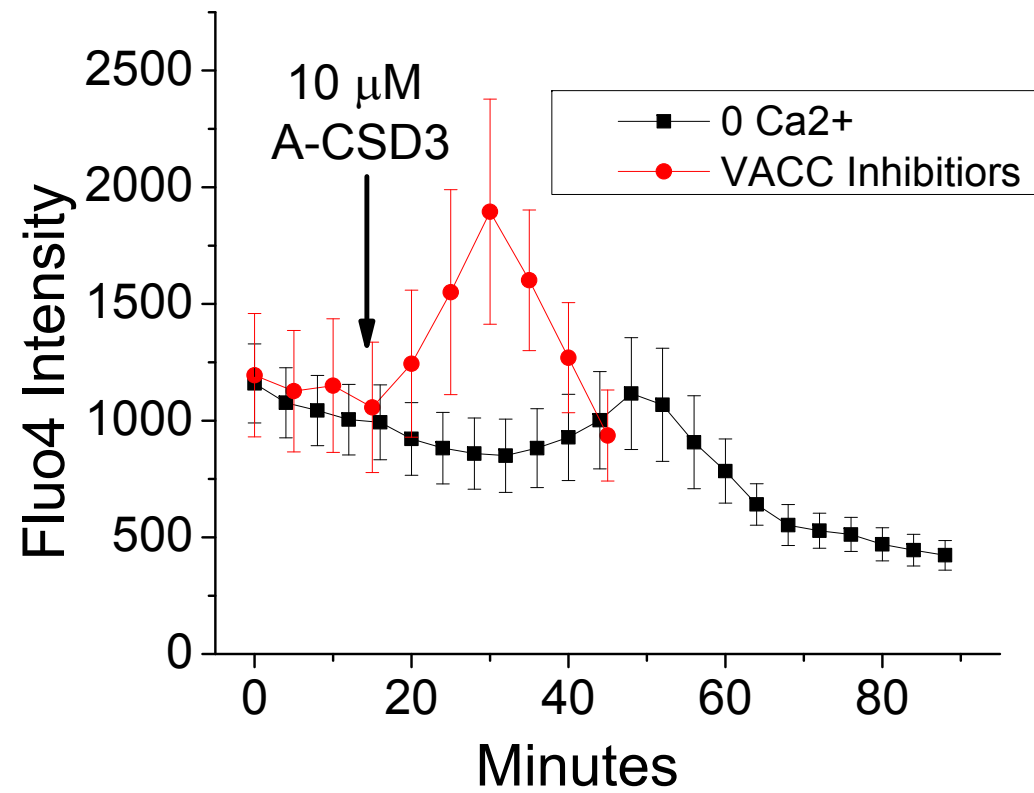

Supporting  
Figure S7

Supplement: Figure S7 — Cav3 associates strongly with unstressed internal membranes. Shows DIC and fluorescent images of an excised patch from a cell expressing Cav3-GFP pressed against coverslip. Cav3-GFP laden vesicles fill the space beneath the dome suggesting Cav3-GFP can readily associate with unstressed membranes. (PDF) [file pone.0072894.s007.pdf]

Cav3-GFP – excised

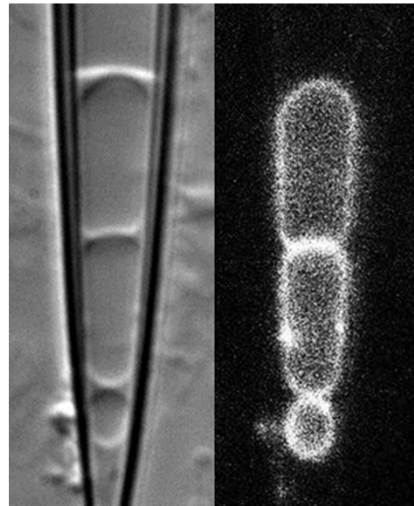

Supporting  
Figure S8

Supplement: Figure S8 — A-CSD3 effect is sensitive to external Ca2+ but not VACC inhibitors. Fluo 4 loaded myotubes were monitored after application of 10 µM A-CSD3 to the bath in the absence of external Ca2+ or in the presence of a cocktail of voltage activated calcium channel (VACC) inhibitors (50 µM Nifedipine, 50 µM Verapamil and 50 µM Diltiazem). VACC inhibitors had no effect on the A-CSD3 induced Ca2+ influx. In the absence of external Ca2+, the response induced by A-CSD3 was delayed and reduced in amplitude. (PDF) [file pone.0072894.s008.pdf]
