# Supplementary material for: Caveolae Regulation of Mechanosensitive Channel Function in Myotubes
Source: PLoS One. 2013 Aug 30;8(8):e72894. doi: 10.1371/journal.pone.0072894 (PMC3758351; doi:10.1371/journal.pone.0072894)
Supplement: Figure S3 — Additional examples of single channel MSC recordings and average currents. (A–C) Show currents as in Figure 2 for three additional patches with different channel conductances and gating kinetics. Each set of recordings shows four representative single channel current recordings at −120 mV membrane potential and conductance recordings at 0 mV membrane potential. Beneath each recording is a red trace showing the average current from 5–15 presure steps. All records were from −70 mmHg stimuli. The 3 recordings show examples of MSC currents from different conductance groups; (A) ∼25 pS channels, (B) ∼50 pS channels, and (C) ∼100 pS channels. The kinetics of the largest conductance channels (C) were very distinct from the lower conductance ones, having shorter open times. (C) Is an example of a patch that likely contains both a low and high conductance MSCs. For example see traces 2 and 3 in the conductance panel indicated by asterisk. (PDF) [file pone.0072894.s003.pdf]

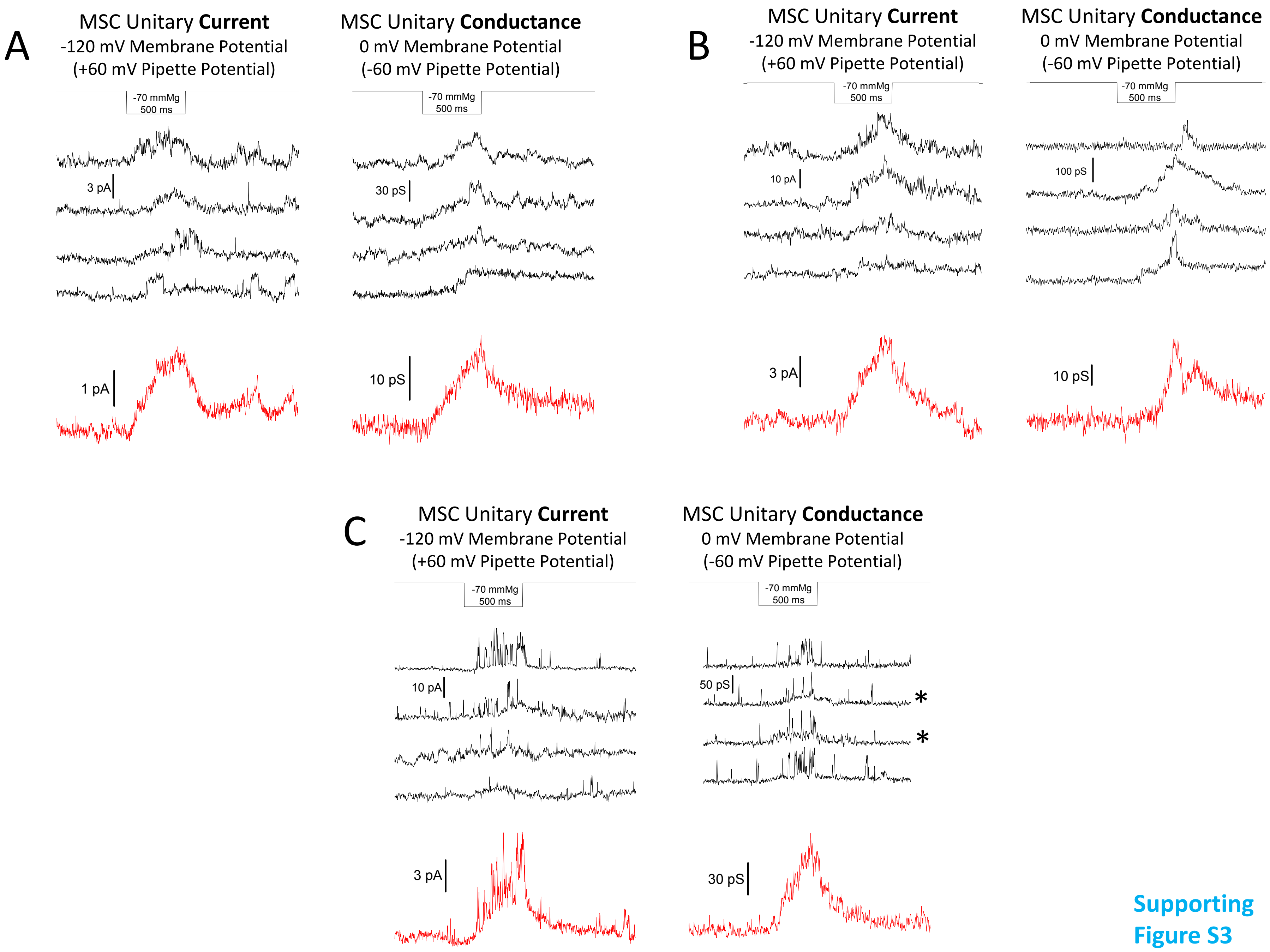

Supporting Figure S3
